# Effect of the Wingman-Connect Upstream Suicide Prevention Program for Air Force Personnel in Training: A Cluster Randomized Clinical Trial

**DOI:** 10.1001/jamanetworkopen.2020.22532

**Published:** 2020-10-21

**Authors:** Peter A. Wyman, Anthony R. Pisani, C. Hendricks Brown, Bryan Yates, Lacy Morgan-DeVelder, Karen Schmeelk-Cone, Robert D. Gibbons, Eric D. Caine, Mariya Petrova, Tracy Neal-Walden, David J. Linkh, Alicia Matteson, Jordan Simonson, Steven E. Pflanz

**Affiliations:** 1 Department of Psychiatry, University of Rochester School of Medicine and Dentistry, Rochester, New York; 2 Department of Psychiatry and Behavioral Sciences, Feinberg School of Medicine, Northwestern University, Chicago, Illinois; 3 Department of Medicine, Biological Sciences, The University of Chicago, Chicago, Illinois; 4 Department of Public Health Sciences, Biological Sciences, The University of Chicago, Chicago, Illinois; 5 Now with Miller School of Medicine, University of Miami, Miami, Florida; 6 US Air Force Surgeon General’s Office, Falls Church, Virginia; 7 Now with Cohen Veterans Network, Silver Spring, Maryland; 8 US Air Force, Head Quarters Air Force, Washington, DC; 9 Now with Syracuse Veterans Affairs Medical Center, Syracuse, New York

## Abstract

**Importance:**

Suicide has been a leading manner of death for US Air Force personnel in recent years. Universal prevention programs that reduce suicidal thoughts and behaviors in military populations have not been identified.

**Objectives:**

To determine whether the Wingman-Connect program for Airmen-in-training reduces suicidal ideation, depression, and occupational problems compared with a stress management program and to test the underlying network health model positing that cohesive, healthy units are protective against suicidal ideation.

**Design, Setting, and Participants:**

This cluster randomized clinical trial was conducted from October 2017 to October 2019 and compared classes of personnel followed up for 6 months. The setting was a US Air Force technical training school, with participants studied to their first base assignment, whether US or international. Participants in 216 classes were randomized, with an 84% retention rate. Data analysis was performed from November 2019 to May 2020.

**Interventions:**

The Wingman-Connect program used group skill building for cohesion, shared purpose, and managing career and personal stressors (3 blocks of 2 hours each). Stress management training covered cognitive and behavioral strategies (2 hours). Both conditions had a 1-hour booster session, plus text messages.

**Main Outcomes and Measures:**

The primary outcomes were scores on the suicidal ideation and depression scales of the Computerized Adaptive Test for Mental Health and self-reports of military occupational impairment. Class network protective factors hypothesized to mediate the effect of Wingman-Connect were assessed with 4 measures: cohesion assessed perceptions that classmates cooperate, work well together, and support each other; morale was measured with a single item used in other studies with military samples; healthy class norms assessed perceptions of behaviors supported by classmates; and bonds to classmates were assessed by asking each participant to name classmates whom they respect and would choose to spend time with.

**Results:**

A total of 215 classes including 1485 individuals (1222 men [82.3%]; mean [SD] age, 20.9 [3.1] years) participated; 748 individuals were enrolled in the Wingman-Connect program and 737 individuals were enrolled in the stress management program. At 1 month, the Wingman-Connect group reported lower suicidal ideation severity (effect size [ES], −0.23; 95% CI, −0.39 to −0.09; *P* = .001) and depression symptoms (ES, −0.24; 95% CI, −0.41 to −0.08; *P* = .002) and fewer occupational problems (ES, −0.14; 95% CI, −0.31 to −0.02; *P* = .02). At 6 months, the Wingman-Connect group reported lower depression symptoms (ES, −0.16; 95% CI, −0.34 to −0.02; *P* = .03), whereas the difference in suicidal ideation severity was not significant (ES, −0.13; 95% CI, −0.29 to 0.01; *P* = .06). The number needed to treat to produce 1 fewer participant with elevated depression at either follow-up point was 21. The benefits of the training on occupational problems did not extend past 1 month. The Wingman-Connect program strengthened cohesive, healthy class units, which helped reduce suicidal ideation severity (estimate, −0.035; 95% CI, −0.07 to −0.01; *P* = .02) and depression symptom scores (estimate, −0.039; 95% CI, −0.07 to −0.01; *P* = .02) at 1 month.

**Conclusions and Relevance:**

Wingman-Connect is the first universal prevention program to reduce suicidal ideation and depression symptoms in a general Air Force population. Group training that builds cohesive, healthy military units is promising for upstream suicide prevention and may be essential for ecological validity. Extension of the program to the operational Air Force is recommended for maintaining continuity and testing the prevention impact on suicidal behavior.

**Trial Registration:**

ClinicalTrials.gov Identifier: NCT04067401

## Key Points

**Question:**

Does group training to build cohesion, shared purpose, and healthy coping for classes of new US Air Force Airmen reduce suicidal thoughts, depression symptoms, and occupational problems?

**Findings:**

In this cluster randomized clinical trial of 1485 personnel in 215 training classes, the Wingman-Connect program reduced suicidal ideation, depression symptoms, and occupational problems at 1 month by fostering cohesive, healthy classes. Reduced depression symptoms were maintained through 6 months, and the odds of having elevated depression symptoms were lower (odds ratio, 0.80) at either follow-up point.

**Meaning:**

Wingman-Connect is the first universal prevention program to reduce suicidal ideation and depression in a general Air Force population.

## Teaser

This cluster randomized clinical trial examines whether the Wingman-Connect program for US Air Force personnel reduces suicidal ideation, depression, and occupational problems compared with a stress management program.

## Introduction

Active duty military suicide rates (24.8 events per 100 000 in 2018)^1,2^ are now comparable to those in the general population, after decades of being lower, prompting unprecedented expansion of suicide prevention activities.^3,4,5^ Military suicide prevention programming has focused on high-risk groups, primarily the detection and treatment of individuals with suicidal thoughts or behaviors.^6,7,8,9^ Yet targeting broad population groups with universal upstream prevention^10^ has similar or greater potential to achieve large reductions in suicide rates.^11,12^ The Department of Defense has prioritized promoting healthy populations to reduce suicides.^2,13^ The US Air Force implemented a multilayered strategy in 1996, which a trend analysis identified as reducing suicides for several years^14^; however, no single intervention was identified as reducing suicide risk.

Universal prevention programs have not been identified that reduce suicidal thoughts and behaviors in military populations. Cognitive skill training impacts physical performance,^15^ social cognition (eg, perspective taking),^16^ coping, and problem solving^17,18^ among US military personnel. Recent testing of coping and interpersonal skill training found no benefits on mental health for Canadian military^19^ or Royal Air Force enlistees^20^; investigators of the Royal Air Force study noted that cognitive training may lack ecological validity without addressing unit functioning.^20^

We developed and then tested Wingman-Connect, a network health suicide and depression prevention program for the US Air Force. Network health interventions^21^ target natural organizational groups to strengthen bonds, cohesion, and adaptive coping norms, all of which are logical targets for upstream military suicide prevention. Similar targets affected in school-based interventions show long-lasting outcomes regarding suicidal ideation and behavior.^22^ Strengthening peer relationships to prevent suicide is consistent with theoretical^23,24^ and empirical evidence showing that disrupted relationships commonly precede military suicides.^2^ Strong bonds reduce vulnerability to depression,^25^ and postenlistment depression is a specific factor associated with suicide attempts among military personnel.^26^ Unit-level influences are major factors associated with suicide risk. Units are foundational structures for military life and health.^27^ Unit cohesion is associated with lower likelihood of suicidal ideation.^28,29^ Data from the Army Study to Assess Risk and Resilience in Servicemembers^27^ showed that up to 20% of suicide attempts over 5 years were associated with membership in a unit in which a member had attempted suicide.

Suicide has been a leading manner of death for Airmen in recent years.^30^ We investigated the Wingman-Connect program as a strategy for strengthening cohesive, protective classes of Air Force personnel in training. Wingman-Connect used a universal prevention strategy^31^ targeting the full population of Air Force trainees across the continuum of risk. The highest suicide rates are among younger enlisted personnel^32^; hence, it was important to focus on personnel entering training. The primary hypothesis tested was that Wingman-Connect would reduce suicidal ideation, depression symptoms, and job-related problems. A secondary objective was testing the guiding network health theoretical model.

## Methods

### Trial Design

All participants provided written informed consent. Study procedures were approved by the institutional review boards of the US Department of Defense and the University of Rochester. Participants were compensated $50 for completing the final 6-month assessment. This study follows the Consolidated Standards of Reporting Trials (CONSORT) reporting guideline for cluster trials. Supplement 1 contains the trial protocol.

**Figure 1.**
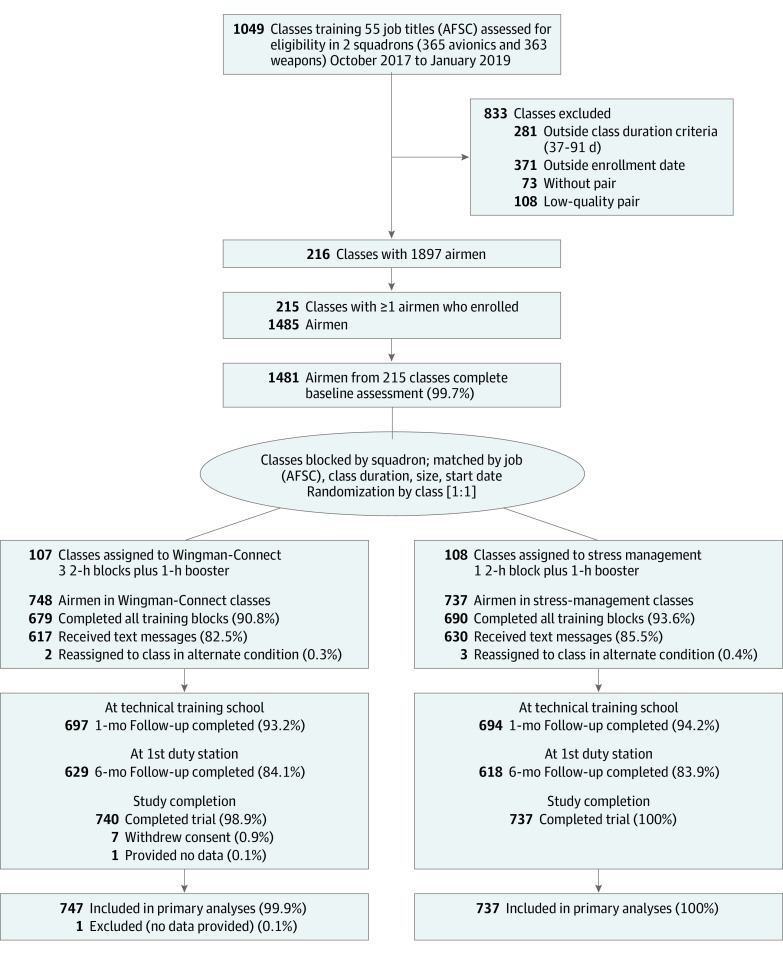
Flow of Technical Training Classes and Airmen in the Wingman-Connect Trial AFSC indicates Air Force Specialty Code.

This cluster randomized clinical trial with head-to-head comparison of 2 conditions^33^ was conducted with new personnel in training assigned to classes at the Technical Training School, Sheppard Air Force Base, Wichita Falls, Texas, between October 2017 and October 2019. We paired similar class units from the same squadron and randomly assigned 1 class from each pair to Wingman-Connect and the other to stress management training (Figure 1). The design used an active comparison training to strengthen internal validity. Matching of classes is described in eAppendix 1 and eTable 1 in Supplement 2.

### Participants

Classes with instructional length between 37 and 91 days were eligible (class size, 4-13 students) (Figure 1). Participants were informed their data would be deidentified at the earliest possible time and that no individual responses would be shared with the Air Force or used for crisis evaluation. All participants received information about mental health resources.

### Interventions

Wingman-Connect was developed between August 2015 and December 2016 at Sheppard Air Force Base by adapting a suicide prevention program used in public education settings (Sources of Strength).^34,35^ For content adaptation, Air Force topic experts identified protective factors essential to an Airman’s job success, supportive of mental health, and theoretically linked to reduced suicide risk. Group training for units was selected to align with Air Force culture, a departure from the Sources of Strength model of training selected opinion leaders.

Wingman-Connect classes participated in group skill building using active learning: high-energy activities and drawing out personal examples occurred in 3 90- to 120-minute blocks over 3 consecutive days. Skills focused on growing and sustaining 4 core protective values: kinship (belonging and accountability), purpose (goals and motivating values), guidance (mentors and institutional resources), and balance (activities for well-being). Each class completed group exercises emphasizing cohesion, shared purpose, and the value of a healthy unit. Participants created videos for class distribution. Peer-nominated participants (2-3 per class) were invited to a 1-hour lunch as training champions (with no formal role). After 1 month, classes were invited to a 1-hour booster review. Training was conducted by 2 trainers with varied backgrounds (educator and mental health clinician) and training (bachelor’s and master’s level) from the research group (1 trainer was L.M.-D.). All Wingman-Connect trainings were audio recorded. Using an established procedure,^36^ coders rated 8 of 22 modules for adherence (mean adherence, 97.7%) (eAppendix 2 in Supplement 2).

If randomized to stress management, participants received an overview of the stress response system and cognitive and behavioral strategies adapted from a cognitive behavioral therapy workbook (2 hours).^37^ Trainers used a slideshow to introduce concepts, show videos, and lead discussions. After 1 month, participants were invited for a 1-hour booster review. Training was conducted by 1 of the 2 trainers from the research team. Participants in both conditions were invited to receive training-specific informational and interactive text messages for 6 months.

### Data Collection

After providing consent, participants completed baseline assessments using a tablet computer and then began their class’s designated training. One month later, after the 1-hour booster session, participants completed the second assessment. Participants provided contact information for the final 6-month assessment, sent via a URL, with up to 4 reminders.

### Outcome Measures

Two of 3 prespecified primary outcomes were the suicide scale (CAT-SS) and depression scale (CAT-DI) of the Computerized Adaptive Test for Mental Health.^38,39,40,41^ The CAT-SS administers a statistically optimal subset of items from a bank of 111 items syndromally associated with suicidal thoughts and behaviors from validated scales (eg, “In last 2 weeks have you felt life is not worth living?”). There is a 52-fold increase in the likelihood of current suicidal ideation—validated against clinician assessment using the Columbia Suicide Rating Scale (C-SSRS)—across the range of CAT-SS scores (0-100, with higher scores indicating worse symptoms).^39^ CAT-SS also yields categorical scores (low, medium, and high); a 1-category change corresponds to a 16 times greater likelihood of an increase in C-SSRS’s ordinal scale, and contrasting high and low risk categories yielded sensitivity of 1.0 and specificity of 0.95 for C-SSRS–rated suicidal ideation.^39^ We combined medium and high categories for this nonclinical sample. CAT-DI uses the same adaptive technology to yield a dimensional measure of depression severity (score range, 0-100, with higher scores indicating worse symptoms); when thresholded (score >35), CAT-DI scores have high sensitivity and specificity for diagnosis of major depressive disorder. Elevated depression and suicidal ideation score dichotomous indicators were created at each follow-up and to indicate an individual scoring above threshold at any point after baseline. See eAppendix 3 in Supplement 2.

The third primary outcome was self-reported behaviorally based performance outcomes for military occupational impairment (eg, corrective training for substandard performance),^42^ summed for a total impairment score (0-5). To supplement this self-report, information on involuntary separations was collected from the squadrons.

Class network protective factors hypothesized to mediate Wingman-Connect impact were assessed with 4 measures. Cohesion assessed perceptions that classmates cooperate, work well together, and support each other (α = .85; 3 items).^43^ Morale was measured with a single item used in other studies with military samples.^44^ Healthy class norms assessed perceptions of behaviors supported by classmates (α = .88; 5 items). Bonds to classmates were assessed by asking each participant to name classmates (up to 5) whom they respect and would choose to spend time with.^45^

Individual factors posited as proximal targets of Wingman-Connect were measured by healthy career behaviors (α = .90; 5 items), help-seeking acceptability (α = .70; 4 items),^35,46^ maladaptive coping (α = .63; 4 items),^47^ personal (α = .63; 2 items) and social problem (α = .83; 3 items) subscales from a measure of military functional impairment,^42^ loneliness,^48^ the anxiety subscale of Computerized Adaptive Test for Mental Health (CAT-AX),^49^ anger intensity and frequency (α = .83; 5 items),^50^ and emotion regulation difficulties (α = .74; 8 items).^51^ Training satisfaction^52^ was assessed at 1 month (α = .91; 3 items).

### Statistical Analysis

The power and sample size estimates were focused on detecting change in the continuous CAT-SS measure. With 1550 participants in 200 classes, expecting 25% attrition, the power and sample size estimates using optimal design showed 80% power with an effect size of 0.15 when the intraclass correlation coefficient was 0.02 and an effect size of 0.17 when the intraclass correlation coefficient was 0.05.

All analyses used an intention to treat approach and included random effects to adjust for class heterogeneity.^53^ All tests used a 2-sided .05 type I error. Baseline equivalence of the randomly assigned groups was tested using multilevel analysis of variance and χ^2^ tests.

We used multilevel linear and logistic mixed-effects regression models in MPlus statistical software version 8 (Muthén and Muthén)^54^ and R statistical software version 4.0.0 (R Project for Statistical Computing)^55^ to test changes due to training separately on 1-month and 6-month outcomes using models controlling for the baseline version of the chosen dependent variable. Our primary analysis included a random intercept to adjust for the nesting of individuals within classes. We also added random effects (intercept and treatment) to account for the nesting of treated and control classes within pairs. Because all of these analyses yielded comparable statistical conclusions and the models with a single random intercept had lower bayesian information criteria, we report findings from that analysis. All models also included covariates of sex, age, race, ethnicity, and active duty vs nonactive duty, all of which have previously been associated with suicide risk.^56^ For CAT dimensional measures, the underlying Bayes estimate of severity was used. For dichotomized scales, 2-level binary models were used. To quantify impact, we calculated Cohen *d* effect sizes (ESs)^57^ for continuous measures and odds ratios (ORs) for dichotomous outcomes. To determine whether Wingman-Connect had a differential impact, we tested baseline by training condition interactions by comparing slopes using Wald type tests that assessed the coefficient’s size to its standard error. We also examined age and sex by training condition interactions. To test the theoretical model positing that a connected, healthy class would be associated with reduced suicidal ideation and depression, we conducted 2-level mediation models using the product of coefficients method^58^; mediators were treated both as individual-level (2-1-1 modeling) and as class contextual effects (2-2-1 modeling).^59^ Formal tests were based on model-based bootstrapped confidence intervals^60^ (see eAppendix 5 in Supplement 2), Data analysis was performed from November 2019 to May 2020.

## Results

### Class and Participant Characteristics

Of 1897 Airmen in the 216 selected classes, 1732 (91.3%) were exposed to their class’s respective intervention, and 1485 participants (85.7%) from 215 classes were enrolled in the study (1222 men [82.3%]; mean [SD] age, 20.9 [3.1] years); 748 individuals were enrolled in the Wingman-Connect program and 737 individuals were enrolled in the stress management program. Exposure and enrollment did not vary by condition. Baseline comparisons accounting for random class effects showed baseline equivalence of the Wingman-Connect and stress management groups (Table 1 and eTable 2 in Supplement 2). A diagnostic P-P plot showed no baseline imbalance (eFigure 3 in Supplement 2). Attrition at 1 month (6%) and 6 months (16%) was not predicted by assigned condition; tests showed no differential attrition. A total of 629 of 748 individuals assigned to Wingman-Connect (84.1%) and 618 of 737 individuals assigned to stress management (83.9%) participated in the 6-month assessment. Training satisfaction scores (range, 0-3) were equivalent in the Wingman-Connect (mean [SD], 1.9 [0.89]) and stress management (mean [SD], 2.0 [0.85]) groups. Fidelity was high for the Wingman-Connect group (98%), and although it was not formally measured for the stress management group, it followed a structured lecture format that ensured high fidelity.

**Table 1.** Baseline Characteristics of the Full Sample, Wingman-Connect Group, and Stress Management Group^a^

Characteristic	Participants, No. (%)^b^		
	Full sample	Wingman-Connect	Stress management
Classes, No.	215	107	108
Squadron			
363 Training squadron weapons	96 (44.7)	48 (44.9)	48 (44.4)
365 Training squadron maintainers	119 (55.3)	59 (55.1)	60 (55.6)
Size, mean (SD), students	6.87 (2.36)	6.93 (2.47)	6.81 (2.23)
Duration, mean (SD), h^c^	533.3 (128.1)	534.8 (128.1)	531.8 (127.5)
Participants, No.	1485	748	737
Sex			
Male	1222 (82.3)	616 (82.4)	606 (82.2)
Female	253 (17.0)	128 (17.1)	125 (17.0)
Age, y			
18	279 (18.8)	140 (18.7)	139 (18.9)
19	359 (24.2)	190 (25.4)	169 (22.9)
20	250 (16.8)	126 (16.8)	124 (16.8)
21-24	410 (27.6)	196 (26.2)	214 (29.1)
≥25	182 (12.3)	94 (12.6)	88 (11.9)
Race			
African American or Black	174 (11.7)	91 (12.2)	83 (11.3)
Asian	62 (4.2)	31 (4.1)	31 (4.2)
Multiracial	131 (8.8)	59 (7.9)	72 (9.8)
Native American or Hawaiian	30 (2.0)	15 (2.0)	15 (2.1)
White	981 (66.1)	498 (66.6)	483 (65.5)
Other	93 (6.3)	47 (6.3)	46 (6.2)
Hispanic or Latino ethnicity	291 (19.6)	137 (18.3)	154 (20.9)
Education			
General education diploma	53 (3.6)	30 (4.0)	23 (3.1)
High school	1167 (78.6)	596 (79.7)	571 (77.5)
AA or AS	159 (10.7)	72 (9.6)	87 (11.8)
BA or BS or higher	101 (6.8)	47 (6.3)	54 (7.4)
Component			
Active duty	1213 (81.7)	617 (82.5)	596 (80.9)
National Guard	182 (12.3)	92 (12.3)	90 (12.2)
Reserve	86 (5.8)	37 (4.9)	49 (6.6)
Prior service	26 (1.8)	15 (2.0)	11 (1.5)

^a^ There were no differences between the Wingman-Connect and Stress Management groups on any variable.

^b^ Some percentages do not equal 100% because of missing data.

^c^ Duration refers to instructional length of the class.

### Impact of Wingman-Connect on Suicidal Ideation, Depression, and Occupational Problems

**Figure 2.**
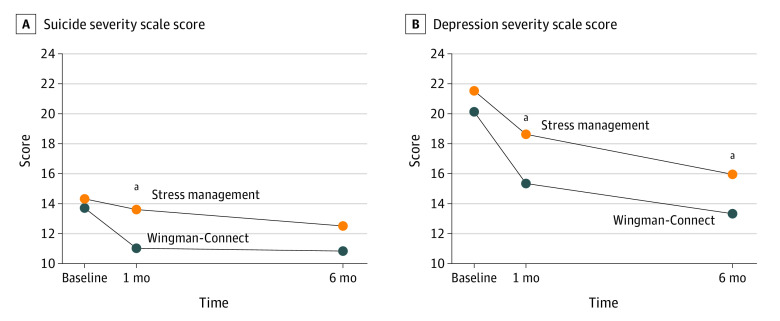
Wingman-Connect and Stress Management Group Scores on Computerized Adaptive Test for Mental Health Graphs show scores for suicidal ideation severity (A) and depression (B) over the 6-month study period. ^a^*P* < .05 for Wingman-Connect vs stress management groups.

The findings showed that the Wingman-Connect program had a beneficial impact on all primary outcomes during technical training (Table 2). Figure 2 shows the mean suicidal ideation scale and depression scale scores for the Wingman-Connect and stress management groups at the 3 assessment points across the 6-month study period.

**Table 2.** Wingman-Connect Impact on Suicide Severity, Depression, and Occupational Impairment (Primary Outcomes) at 1-Month and 6-Month Follow-up

Variable	Score, Mean (SD)						Impact^a^			
	Wingman-Connect (n = 747)			Stress management (n = 737)			1-mo follow-up		6-mo follow-up	
	Baseline	1 mo	6 mo	Baseline	1 mo	6 mo	ES (95% CI)	*P* value	ES (95% CI)	*P* value
Suicidal ideation severity	13.72 (12.97)	11.05 (13.25)	10.76 (13.87)	14.32 (13.34)	13.43 (13.94)	12.12 (13.50)	−0.23 (−0.39 to −0.09)	.001	−0.13 (−0.29 to 0.01)	.06
Depression symptoms	20.11 (15.76)	15.42 (16.93)	13.36 (17.18)	21.60 (16.81)	18.38 (16.87)	15.64 (15.21)	−0.24 (−0.41 to −0.08)	.002	−0.16 (−0.34 to −0.02)	.03
Occupational impairment^b^	0.27 (0.65)	0.17 (0.62)	0.29 (0.93)	0.32 (0.72)	0.25 (0.73)	0.28 (0.94)	−0.14 (−0.31 to −0.02)	.02	0.01 (−.12 to .11)	.82
Elevated scores, participants, No. (%)^c^^,^^d^										
Suicidal ideation severity	47 (6.3)	50 (6.7)	43 (5.8)	67 (9.1)	59 (8.0)	52 (7.1)	0.91 (0.70 to 1.21)^e^	.25	0.89 (0.67 to 1.19)^e^	.22
Depression	119 (15.9)	83 (11.1)	62 (8.3)	151 (20.5)	109 (14.8)	83 (11.3)	0.78 (0.64 to 0.97)^e^	.01	0.82 (0.63 to 1.05)^e^	.07

Abbreviation: ES, effect size.

^a^ Negative regression estimates and ESs indicate beneficial Wingman-Connect impact. All models were adjusted for class (random effect) and sex, age, race/ethnicity, and Air Force component.

^b^ Wingman-Connect benefit at 1 month was greater for personnel with more occupational problems at baseline, as evident by training condition × baseline interaction (relative change, −0.38; 95% CI, −0.78 to −0.08). No other baseline × treatment condition interactions were significant.

^c^ A suicide scale score greater than 34 was considered elevated. Elevated scores for suicidal ideation at 1 and/or 6 months were present among 77 participants (10.3%) in the Wingman-Connect group and 93 participants (12.6%) in the stress management group (odds ratio, 0.81; 95% CI, 0.61 to 1.07; *P* = .07; number needed to treat [ie, training in Wingman-Connect to reduce 1 person with elevated risk], 44).

^d^ A depression scale score greater than 35 was considered elevated. Elevated depression scores were present in 120 participants (16.1%) in the Wingman-Connect group and 154 participants (20.9%) in the stress management group (odds ratio, 0.80; 95% CI, 0.64 to 0.97; *P* = .01; number needed to treat, 21).

^e^ Data are odds ratio (95% CI).

At the 1-month follow-up, participants in Wingman-Connect classes reported lower suicidal ideation severity (ES, −0.23; 95% CI, −39 to −0.09; *P* = .001) and depression symptoms (ES, −0.24; 95% CI, −0.41 to −0.08; *P* = .002) and fewer occupational problems (ES, −0.13; 95% CI, −0.31 to −0.02; *P* = .02). The odds of elevated depression were 22% lower for participants in the Wingman-Connect group compared with the stress management group (OR, 0.78; 95% CI, 0.64 to 0.97). Wingman-Connect participants were less likely to report corrective training (OR, 0.51; 95% CI, 0.31 to 0.82) or receiving a negative counseling statement (OR, 0.50; 95% CI, 0.29 to 0.86) compared with participants in the stress management group (eFigure 1 in Supplement 2). Squadron data were consistent with participants’ self-reports: 7 participants in the stress management group separated from the Air Force compared with 4 participants in the Wingman-Connect group.

At 6-month follow-up, Wingman-Connect participants reported significantly lower depression symptoms (ES, −0.16; 95% CI, −0.34 to −0.02; *P* = .03) (Table 2), whereas suicidal ideation severity scores were not significantly lower (ES, −0.13; 95% CI, −0.29 to 0.01; *P* = .06). A beneficial impact on occupational problems was not evident after 6 months. Wingman-Connect participants were 20% less likely than participants in the stress management group to report elevated depression at either follow-up point (OR, 0.80; 95% CI, 0.64 to 0.97; *P* = .01) and 19% less likely, on average, to report elevated suicidal ideation scores, although the difference was not significant for suicidal ideation scores (OR, 0.81; 95% CI, 0.51 to 1.07; *P* = .07) (Table 2). The number needed to treat to produce 1 fewer participant with elevated depression at either 1 or 6 months was 21.

The analyses showed no differential effects of Wingman-Connect on suicidal ideation severity (1 month) or depression symptoms (1 month and 6 months) by levels of those problems at baseline, or by sex or age (ie, no moderation). The Wingman-Connect reduction in occupational problems at 1 month was greater for trainees who had more problems at baseline (Table 2).

### Impact of Wingman-Connect on Hypothesized Mediators

Wingman-Connect participants gained on all class protective factors: cohesion, morale, bonds to classmates, and perceptions that members support healthy behaviors (ES, 0.18 to 0.23) (Table 3). Wingman-Connect increased positive career behaviors (ES, 0.16; 95% CI, 0.02 to 0.28) and reduced anxiety (ES, −0.14; 95% CI, −0.29 to −0.01) and anger (ES, −0.18; 95% CI, −0.35 to −0.04). Social (ES, −0.23; 95% CI, −0.49 to −0.02) and personal (ES, −0.54; 95% CI, −0.93 to −0.23) impairments were reduced among participants with the highest third of problems at baseline. Training group differences were not found regarding coping attitudes, loneliness, or emotion dysregulation.

**Table 3.** Wingman-Connect Impact on Targeted Class-Individual Risk and Protective Factors in Technical Training^a^

Measure	Score, Mean (SD)				ES (95% CI)	RC (95% CI)
	Wingman-Connect		Stress management			
	Baseline	1 mo	Baseline	1 mo		
Class characteristics						
Class cohesion^b^	3.12 (0.59)	3.16 (0.72)	3.09 (0.58)	3.05 (0.75)	0.18 (0.04 to 0.29)^c^	−0.10 (−0.35 to 0.11)
Class morale^b^	3.78 (0.91)	3.79 (0.96)	3.68 (0.90)	3.63 (1.00)	0.23 (0.05 to 0.40)^c^	0.03 (−0.13 to 0.17)
Healthy class norms^b^	2.95 (0.55)	3.10 (0.63)	2.90 (0.57)	3.01 (0.62)	0.18 (0.04 to 0.30)^c^	−0.22 (−0.53 to 0.04)
Bonds to classmates^b^	2.31 (1.65)	2.08 (1.61)	2.07 (1.62)	1.83 (1.56)	0.21 (0.05 to 0.35)^c^	−0.03 (−0.11 to 0.04)
Individual characteristics						
Healthy career behaviors	1.72 (0.61)	1.69 (0.75)	1.70 (0.64)	1.60 (0.72)	0.16 (0.02 to 0.28)^c^	−0.11 (−0.32 to 0.08)
Help seeking acceptability	3.10 (0.56)	3.18 (0.62)	3.12 (0.57)	3.12 (0.61)	0.12 (−0.01 to 0.23)	−0.06 (−0.30 to 0.16)
Maladaptive coping attitudes^d^	1.61 (0.46)	1.63 (.049)	1.62 (0.47)	1.64 (0.50)	0.00 (−14 to 11)	0.07 (−0.25 to 0.34)
Military functional impairment^d^						
Social	0.62 (0.64)	0.51 (0.68)	0.57 (0.66)	0.56 (0.70)	−0.10 (−0.26 to 0.04)	−0.23 (−0.49 to −0.02)^c^^,^^e^
Personal	0.35 (0.57)	0.34 (0.62)	0.35 (0.58)	0.40 (0.69)	−0.10 (−0.24 to 0.03)	−0.54 (−0.92 to −0.23)^c^^,^^e^
Loneliness^d^	1.80 (0.76)	1.73 (0.77)	1.73 (0.74)	1.78 (0.73)	−0.10 (−0.26 to 0.05)	−0.03 (−0.19 to 0.11)
Anxiety^d^	10.95 (13.86)	9.91 (15.55)	12.40 (15.94)	11.55 (15.75)	−0.14 (−0.29 to −0.01)^c^	0.10 (−0.14 to 0.31)
Anger^d^	0.53 (0.66)	0.43 (0.62)	0.55 (0.65)	0.51 (0.66)	−0.18 (−0.35 to −0.04)^c^	−0.31 (−0.67 to 0.00)^c^^,^^e^
Emotion regulation difficulties	1.91 (0.62)	1.92 (0.69)	1.91 (0.61)	1.95 (0.65)	−0.07 (−0.23 to 0.08)	0.00 (−0.20 to 0.19)

Abbreviations: ES, effect size; RC, relative change.

^a^ All models were adjusted for class (random effect), sex, age, race/ethnicity, and service component. RC refers to the training condition × baseline interaction and shows the Wingman-Connect vs stress management impact per 1 unit difference at baseline on that variable. ESs are main effects without baseline × training condition interaction in model.

^b^ Indicates that this measure loads on the Connected Thriving Class factor used in mediation analysis.

^c^ Indicates that ES and RC (95% CI) are significant (*P* < .05).

^d^ Higher scores on these measures indicate greater risk; therefore, negative regression estimates and ESs indicate beneficial impacts of Wingman-Connect.

^e^ For participants in the highest tercile of problems at baseline, Wingman-Connect was associated with significantly reduced social functional impairment (ES, −0.27; 95% CI, −0.51 to −0.08), personal functional impairment (ES, −0.30; 95% CI, −0.53 to −0.11), and anger (ES, −0.30; 95% CI, −0.57 to −0.09) vs stress management.

### Testing Mediational Pathways: Connected Healthy Class to Reduced Suicidal Ideation and Depression at 1 Month

We constructed a latent factor—cohesive healthy class networks—using all class variables with excellent fit with the data (eAppendix 4 in Supplement 2). To test mediation, we ran models that separately estimated 1-month suicidal ideation and depression scores using all previously reported covariates, baseline outcome scores, and training conditions, and then added both the baseline and 1-month latent class factor. We did not find significant mediation using the class-level mediator for each individual. Detailed findings for the individual level mediation (2-1-1 model) are shown later. The formal test of mediation—based on the product of coefficients in the indirect paths from condition to class factor and from class factor to 1-month suicidal ideation and depression scale scores—showed significant mediation for suicidal ideation (estimate, −0.035; 95% CI, −0.07 to −0.01; *P* = .02) and for depression symptoms (estimate, −0.039; 95% CI, −0.07 to −0.01; *P* = .02) (see eFigure 2 in Supplement 2). Participants’ perceptions of being embedded in a more-cohesive, healthy class accounted for significant portions of Wingman-Connect’s impact on reducing suicidal ideation and depression symptoms.

## Discussion

Wingman-Connect is the first universal upstream prevention program tested through a randomized clinical trial to reduce suicidal ideation and depression symptoms in a general, nonclinical Air Force population. The magnitude of effects at 1 month (ES, −0.23 to −0.24) was equivalent to those of state-of-the-art prevention programs targeting broad adolescent and young adult populations where the majority are not at high risk.^61^ The beneficial impact on reduced depression symptoms was maintained at 6 months, including lower likelihood of elevated depression (OR, 0.80) over the full study period. The effects of Wingman-Connect in reducing suicidal ideation severity (1 month) and depression symptoms (1 and 6 months) were distributed across personnel with different levels of those problems at baseline. That diverse personnel benefited from the program illustrates a strength of a universal prevention strategy for military populations with members at low risk and others at higher risk who may not seek mental health services.

Wingman-Connect–trained personnel reported fewer occupational problems at 1 month (ie, negative counseling statement [OR, 0.50] or corrective training [OR, 0.51]). The dual benefits for occupational functioning and mental health underline a strength of upstream prevention implemented before the detection of serious suicidal behavior: skills that strengthened the trainee’s capability to meet job-related challenges also reduced depression and suicidal ideation. Universal prevention programs that support operational and suicide prevention objectives are more likely to be sustained.

The study’s findings validate the underlying network health model: stronger bonds within a more cohesive healthy class reduced suicidal ideation and depression symptoms.^21^ These findings suggest that Wingman-Connect classes became increasingly unified around healthy norms and encouraged classmates who were vulnerable to mental health or occupational problems at a key juncture of military training, in addition to meeting their needs for belonging.^23,24^ Work beginning with Durkheim plus recent social network modeling^45^ show that cohesive groups serve a protective regulatory function through norms and pressures to conform.^62,63,64^ Future studies should also test whether Wingman-Connect would be less effective with Air Force personnel who are minimally connected to their assigned class.

An innovation of Wingman-Connect was training in natural organizational units. The positive outcomes suggest that Wingman-Connect leveraged the influence that personnel have on each other daily. Half of the training was dedicated to class skill building. This peer-to-peer approach differs from most current suicide prevention trainings. Group training for units may be critical for ecological validity of military suicide prevention programs^20^ and for transfer of skills into the organizational culture.

Expansion of the Wingman-Connect program to active duty Air Force bases will likely be necessary to promote protective working units for continuity of its impact. A reduction in occupational problems was not sustained beyond technical training. Future studies are needed to determine whether the Wingman-Connect program is associated with prevention of suicidal behaviors, which personnel benefit most, and what degree of saturation of Wingman-Connect–trained personnel will optimize its impact.

### Limitations

Study limitations include the fact that participants and school leadership were not blinded to the intervention condition. Although training satisfaction ratings were comparable between the 2 conditions, the impact of awareness of the condition on self-ratings cannot be ruled out. Second, the outcomes relied on self-reports; however, these self-reported measures have been validated against structured clinical interviews with strong agreement. Third, trainers were research staff, and the findings may not generalize to training by military personnel.

## Conclusions

In this cluster randomized clinical trial, the Wingman-Connect program reduced suicidal ideation, depression symptoms, and occupational problems for Airmen in technical training (at 1 month of follow-up), and the benefits on reduced depression symptoms were sustained for 6 months. Further research is recommended to test upward extension into operational Air Force and longer-term impact on preventing suicidal behaviors.

## Notes

Supplement 1. **Trial Protocol**

Supplement 2. **eAppendix 1**. Selection and Matching of Technical Training Classes **eTable 1. **Matched Pairing of Enrolled Technical Training Classes **eAppendix 2.** Procedures for Rating Adherence to Wingman-Connect Training Manual **eAppendix 3.** Modification of CAT-SS Modification of CAT-SS Item Bank by Removing 1 Item **eAppendix 4**. Protocol Deviations in Statistical Analyses **eAppendix 5.** Statistical Analyses **eTable 2.** Baseline Outcomes and Risk/Protective Factors for Full Sample, Wingman-Connect Group and Stress Management Group **eFigure 1.** Wingman-Connect Impact on Occupational Impairment Indicators in Technical Training **eFigure 2.** Wingman-Connect Impact on Suicide Severity and Depression Mediated Through Stronger Class Units **eFigure 3.** P-P Plot of Tests of Baseline Equivalence on Behavior Measures **eReferences.**

Supplement 3. **Data Sharing Statement**
