# Supplementary material for: Effect of the Wingman-Connect Upstream Suicide Prevention Program for Air Force Personnel in Training: A Cluster Randomized Clinical Trial
Source: JAMA Netw Open. 2020 Oct 21;3(10):e2022532. doi: 10.1001/jamanetworkopen.2020.22532 (PMC7578767; doi:10.1001/jamanetworkopen.2020.22532)
Supplement: Supplement 1. — Trial Protocol [file jamanetwopen-e2022532-s001.pdf]

Study Title: Career Enhancement Training Study (US-AF)

Principal Investigator: Peter A. Wyman, PhD
Department of Psychiatry, University of Rochester

Co-Investigators: Anthony R. Pisani, PhD
Eric D. Caine, MD
Department of Psychiatry, University of Rochester

C Hendricks Brown, PhD
Northwestern University
Robert Gibbons, PhD
University of Chicago

1. Abstract: Project Objectives/Aims

Relationship disruptions and social isolation are major precipitants for military suicides and a range of problems that impair functioning and reduce mission readiness (DoD, 2012). However, nearly all current military suicide prevention programming focuses on a narrow range of approaches for individuals already at high-risk (e.g., training to detect warning signs and refer for treatment services). This project addresses the need for universal prevention approaches to proactively strengthen protective factors across military populations, including strengthening social connectedness and skills to grow and sustain relationships across service.

The aims of this project are to (a) adapt the Sources of Strength intervention (Wyman et al., 2010) for young, enlisted United States Air Force personnel, and (b) test the adapted intervention (i.e., Wingman-Connect) using a rigorous randomized controlled trial (RCT) design. Sources of Strength has proven efficacy in strengthening social integration, social support interactions, and healthy orientation to managing stressors such as relationship break-ups that increase vulnerability to emotional problems including suicidal behavior (Wyman et al., 2010). Those gains spread across a population in education settings to untrained individuals (Wyman et al., 2010; Petrova, Wyman, Schmeelk-Cone, & Pisani, 2015).

This project was planned as three phases: adaptation and development (Phase 1); field test to assess acceptability and feasibility of the adapted intervention (Phase 2); and randomized controlled trial with 6-month follow-up (Phase 3). We have completed Phase 1 through a period of curriculum development with Airman-in-Training (AiT) at the 82nd Training Wing (82 TRW, Sheppard Air Force Base) from August 2015 – February 2017. Due to the extended Phase 1 (approximately 16 months beyond the initially planned duration), the project leadership team (including Air Force partners) determined that the 'field test' phase is unnecessary.

In Phase 1 we enrolled a total of 296 Airman-in-Training (from 29 technical training classes) in 10 cohorts over 18 months to pilot and evaluate new training modules aimed at strengthening social connectedness and coping resources. Participants completed assessments of targeted constructs (e.g., class cohesion, help-seeking acceptance) prior to training and again at 1-month follow-up, as well as measures of knowledge retention and preparation to apply skills. Rates of training retention (completing all 5 hrs. of training) were also collected for each cohort. Data were used to revise and improve training modules and evaluate progress of the adaptation. Data collected during Phase 1 suggest that Wingman-Connect training is engaging AiTs, the concepts are retained, students view the training as beneficial, and those indicators have improved over time. Retention of core concepts (active recall) was 20% higher in the last several cohorts trained (reaching nearly 100% at the end). More recent versions of the training show increased gains in class cohesion over time. Class cohesion change scores (pre-training to 1-month follow-up) doubled in size after cohort 4 (0.5 SD to 1.0 SD).

In Phase 2, we will conduct a cluster randomized controlled trial to compare the completed Wingman-Connect intervention with a stress management training. Subjects will be approximately 1550 Airmen-in-training (AiTs) attending technical school at Sheppard Air Force Base (82 TRW) will participate as research subjects. We project 15-20% attrition, resulting in approximately 1200 participants completing the follow-up (6-months post-training). Random assignment will occur at the class level, since Wingman-Connect is intended for members of a class together as a unit.

The primary aims will test the following hypotheses:

1. AiTs in Wingman-Connect trained classes compared to controls will report lower suicidal ideation, depression and occupational fitness problems. Determine if differences on outcomes vary by baseline level of problems, and by demographic characteristics.
2. AiTs in Wingman-Connect trained classes will report higher connectedness to classmates and healthier perceived class norms; more adaptive help-seeking norms, fewer problems with anger, loneliness, and difficulties adjusting to military environment.
3. A secondary objective is to test mediators. We will test the theoretical model underlying Wingman-Connect, that reductions in suicidal ideation and depression occur when more healthy relationship ties are created and strengthened among those in the same workforce group. A second mediator is hypothesized to be improved emotion regulation, from gains in coping resources and norms taught through Wingman-Connect.

2. Characteristics of the Research Population and Study Site

2.1 Subject Characteristics. Based on Air Force administrative databases regarding AF personnel, the demographics of the 1550 subjects are projected to approximate the overall AF active-duty population: 19% female (n=295) and 81% male (n=1255). We project that approximately 78% of personnel can be expected to be White (n=1209), 20% Black (n=310), and 2% Asian (n=31). The proportion of other race groups (e.g., Native American; Alaskan Native) is not known. Based on estimates from the Air Force Personnel Center (http://www.afpc.af.mil/library/airforcepersonneldemographics.asp) approximately 7% (n=109) are projected to self-identify as Hispanic ethnicity. All subjects will be 18 years or older, with expected range of 18 – 35. Based on Air Force recruiting standards, all participants are expected to have completed high school, partial college or college degree (2- or 4-year), and possess English language proficiency required for informed consent.

2.2 Research Site. All subjects and training will be located in two squadrons of the 82nd Training Wing (Sheppard Air Force Base, Wichita Falls, Texas): the 365th TRS and the 363rd TRS. Letters of participation from the squadron commanders are attached to this Phase 2 protocol.

2.3 Inclusion/Exclusion Criteria. Airmen-in-Training (AiT) in Technical Training classes in the 365 or 363 Training Squadrons of the 82 Training Wing (82 TRW) will be eligible to participate. There are no restrictions based on age, sex, race/ethnicity or any other personal characteristic. All AiTs are expected to have sufficient English language proficiency for training and standardized assessments. By enrolling AiTs in two training squadrons, 365 and 363, study participants will reflect diversity of job classifications. The 365 trains Airman primarily in avionics (electronics systems), and the 363 trains in weapon systems. Overall, more than 40 different job classifications (AFSCs) are trained across both squadrons.

Both intervention conditions (Wingman-Connect and Stress Management Training) have a brief text messaging component. Owning a phone and using text messaging is not required for participation in the study.

Classes will be selected to receive information about the study (time and place of informational meetings) that fit in a ‘window’ of instructional length 37 – 91 days. Specifically, classes will receive study information that have a training duration long enough for participants to complete the 1-month follow-up assessment while still enrolled in training program at Sheppard Air Force Base (pre-graduation) and short enough so that participation in the follow-up assessment will occur 2-4 months after transfer to first duty base. In the 363rd eligible classes will be those with 8 – 12 week duration; in the 365th, 16 – 25 week duration, which includes a 7 week fundamentals class prior to entering their specific AFSC technical class and being eligible to enroll. Classes will be invited to enroll in the first 2 – 8 weeks of starting their AFSC technical training class.

Baseline assessments will be completed after enrollment through an on-line system (see below). Individual characteristics are not examined prior to enrollment, and there are no exclusion criteria due to any self-reported mental health symptom or other attribute.

3. Study Personnel, Design, Recruitment, and Consent

3.1. Study Personnel and Roles. University of Rochester Medical Center (URMC) personnel will conduct all subject recruitment, enrollment, in-person surveys, and training at the study site (Sheppard Air Force Base). No other study personnel (from Northwestern University or University of Chicago) will have direct contact with subjects or access to identifiable subject data.

3.2. Cluster Randomized Trial Design. The study design will involve randomizing approximately 1550 Airman-in-Training within 160 Technical Training classes to one of two conditions: Wingman-Connect or a brief Stress Management

training. Randomization will occur at the class level, since Wingman-Connect is designed to train AiTs within a class together as a group (those who voluntarily enroll). We have designed this trial to stagger recruitment and training of the different groups over the course of approximately 14-16 months in a "roll-out" randomized trial design (Brown et al., 2009). In this design, we will form clusters of class units within training squadron in order to balance the two conditions on key characteristics including age, job classification and time of year. We have documented that such designs that randomize across "place and time", i.e. workforce group and cohort, have both beneficial statistical properties and can be conducted effectively in large systems (e.g. schools and county service organizations) (e.g., Wyman, Brown et al., 2008).

In the randomly assigned control condition, AiTs will receive a 2-hr training on stress management (Stress Management Training). Participants in both Wingman-Connect and Stress Management conditions will be sent brief informational text messages. Comparing Wingman-Connect with an alternative training is a stronger design than using a no-intervention condition, by increasing the extent to which the design 'controls' for potential impact that could come from individual's knowledge that he/she is participating in a training designed to improve well-being and from receiving messages about how to enhance personal well-being, particularly for isolated personnel.

3.3. Method of Subject Identification and Recruitment. We will use procedures developed for Phase 1 designed to optimize informed consent and eliminate any potential pressure that AiTs could perceive to participate in the study.

UR research staff will identify 6-12 eligible technical training classes on a monthly or bi-monthly basis made up of 60 – 70 AiTs (frequency depending on availability within squadron), from training registries obtained from the Sheppard AFB registrar (which will only list classes, no names or other identifiers). The Squadron staff will confirm eligibility of proposed classes and provide those AiTs with information on the time and place that the researchers will be available if they wish to learn about the study, during a 'non-restricted' portion of the training day. The researchers will be at a different location than leadership (Commander, Supervisors, Instructors), and the recruitment will occur at that location. No leadership (of the squadron or other group) will be present during the recruitment. The leadership will not encourage participation in the study, and the researchers will not provide any information to leadership about whether students enrolled. Each AiT will be introduced to the study and can decide whether to participate. Research staff presenting to AiT groups for recruitment will clearly state that participation in the study is voluntary. They will be informed that they can decline to participate in the research study and, if they do choose to participate in the research, to withdraw their participation at any time.

3.4. Process of Consent. Each prospective participant will receive a consent form outlining all required elements. A trained URMC research staff member will summarize the key elements of the consent document, including voluntary nature of the study; rights of subjects to withdraw at any time; the purpose and procedures of the study, including the types of information and data being collected, and random assignment into one of two trainings (Wingman-Connect, or Stress Management); potential risks and steps that will be taken to address those risks, and limits of confidentiality of data. During the consent form review, research staff will inform potential subjects that individual responses to questionnaires covering behavioral and emotional health (including the measures of mood and distress) will be de-identified at the earlier possibly opportunity and not examined for the purpose of identifying individuals for referrals. All individuals will receive information about available mental health resources (including external help lines). URMC research staff will inform prospective participants that enrollment requires providing an email address and/or a mobile phone number, so that participants can be sent information about how to complete the 6-month follow-up assessment. Potential subjects will be informed that assessments will be completed through a secure on-line platform, and ID codes linking each subject to his/her data will be de-identified before the data is analyzed.

Time will be allotted for participants to read the consent documents and ask questions of qualified research staff. Individuals who choose to enroll as subjects will provide signed a consent document to research staff (who will sign to document obtaining consent and having provided opportunity for the subject to ask questions). Because participation in the study is minimal risk and involves training on knowledge and skills to (a) build and sustaining healthy relationships and social networks (Wingman-Connect), or (b) understand stress and how to manage/reduce stress (Stress Management), a formal 'check' of understanding of the study procedures/elements is not necessary. All subjects are expected to be English-proficient, which is a requirement of being active-duty AF personnel. Subjects will not receive payment for participation while they remain in training. Per recommendation of Sheppard Air Force Base Leadership, all training and assessments will be conducted during non-restricted portions of their duty day, during which airmen may not receive extra compensation. Potential subjects will be informed that they will be eligible for a $50 honorarium (gift card) for completing the final 6-month follow-up assessment during off-duty hours, after transfer to their first duty base. Participants will also be given the option to decline the honorarium, based on recommendations from the PI of a large military cohort study, which found that some service members prefer to forego payment (personal communication, Dennis Faix, MD, MPH, Director, Military Population Health, Naval Health Research Center). Participants will be asked to provide a primary and back-up electronic contact (phone

or email) in order to be contacted for the final survey and payment. This information will be stored separately from participant's research data and will be deleted at the completion of the study.

At the time subjects provide written consent, each subject will be given a second copy of the consent form for his/her records, which includes information about how to contact the researchers and RSRB at University of Rochester. Subjects will be asked to sign both copies. Photocopying machines may not be routinely available, so copies of signed consent forms may not be available to provide subjects, and it is viewed as more important that subjects receive a copy immediately for contact information.

3.5. Process of Randomization. Randomization will occur at the Technical Training Class level, since Wingman-Connect is designed to promote skills of individuals as well as cohesion at the unit (class) level. Following squadron leadership identifying eligible classes of 60-80 AiTs, the classes will be paired and randomly assigned using a random number generator to either Wingman-Connect or Stress Management Training condition. Classes will be paired within squadron. By pairing Technical Training Classes within the same squadron that begin instruction at approximately the same period in time, our design reduces the potential for external events (e.g., leadership change, violent incident on base) biasing students' behavior or self-reports within one of the conditions, which could reduce our ability to evaluate intervention impact (Brown et all., 2006). To conduct the randomization, class numbers will be sent to Northwestern University site PI (C Hendricks Brown, PhD) who will conduct the randomization using programmed random number generator. No names or identifying information about the class will be sent, just an ID code (for class).

AiTs who enroll will complete a baseline assessment (see below for measures). After completing the assessment, participants will be informed of the time and location of the training to which they are assigned. Training will also occur during non-restricted portions of the day.

4. Training Procedures/Conditions

4.1 Training. Groups of 25-35 subjects will participate in either (a) **Wingman-Connect**, or (b) **Stress Management Training** based on random assignment.

4.2 Wingman-Connect Training. Wingman-Connect was created by adapting the evidence-based Sources of Strength program (LoMurray, 1995; Wyman et al., 2010) for the US Air Force. Sources of Strength has proven efficacy in strengthening social integration, social support interactions, and healthy orientation to managing stressors, and those gains spread to untrained individuals (Wyman et al., 2010; Petrova, Wyman, Schmeelk-Cone & Pisani, 2015). Wingman-Connect total training time is 5 hours, typically spread over three consecutive training 'blocks'(or days), plus 1-hour of booster training (one month later).

Wingman-Connect training focuses on strengthening skills to grow and sustain four protective factors: Kinship (healthy bonds); Guidance (support from mentors; access to medical and mental health expertise); Purpose (goals, sense of being valued and valuable); Balance (self-care and support) (Four Cores). Trained facilitators, who apply an evidence-based active training model developed through training thousands of young adults and adolescents in Sources of Strength, lead interactive modules to draw out "real world" narratives of healthy bonds and coping as the primary method of teaching about the Four Core protective factors. The training has a total of 22 modules comprised of specific learning objectives and activities. Key modules and associated skills include: eliciting reasons for enlisting as a motivational foundation for the training; introducing Four Cores as protective factors that promote fitness, well-being and preparedness to benefit from AF career opportunities; elicit self-reflection on participants' own balance of Cores and ways to strengthen each Core; establishing the importance of personal and career success as depending on a strong network; extending learning into natural environment by inviting classes to work together to strengthen collective kinship, purpose, guidance or balance; establishing norm that AiTs are strengthening Cores and consolidating gains by drawing out narratives about how participants' are keeping one or more Cores strong.

Between 1st and 2nd training block approximately two AiTs per class (peer nominated) are invited to attend voluntary 1-hr 'Key Wingman' lunch (during unrestricted time) to learn how they can be positive influencers for the training (no formal role or requirements). After 1 month, classes are invited for a 1 hr. booster with seven modules aimed at reviewing and consolidating accomplishments and strategies to overcome barriers to re-establishing strong Cores after transition out of technical training. These "Key Wingman" are selected by nominations of classmates on the baseline social network measure (participants name classmates who they trust and would choose to spend time with). The 1-hr 'Key Wingman' session has modules designed to empower this group with knowledge that they are seen by their peers as influential; provide them experience of articulating their experiences in growing Four-Core protective factors so they can model healthy narratives to others and serve as positive role models during the training. No additional data is collected from Key Wingman and no

additional activities are required of those individuals outside of usual Wingman-Connect training that would require additional consent procedures (standard consent forms describe nomination, voluntary additional meeting).

A core concept of Wingman-Connect is that individuals sharing real-world examples of strength helps create a healthy culture. Participants in the training will therefore be invited to contribute to two optional video-recorded *public service campaigns*. These strength-based campaigns provide opportunities for participants to share a brief "testimonial" about how they maintain and strengthen supportive relationships and other "Four-Cores" of strength. Participants will be informed at the time they are given the option to participate that these strength-based videos: are voluntary; will be sent out to participants in their training cohort (email, text); and will be available to share with other Airmen and family outside the training group via a weblink on Wistia.com. These digital recordings will not contain any sensitive information (all video recordings are reviewed by the PI). These public service campaign videos do not identify anyone as a study participant, could not be used to link any individual to study data, and will be not be retained as data.

After 1 month, Wingman-connect has a 1 hr. "Booster" session. The Booster has seven modules aimed at reviewing and consolidating accomplishments and strategies to overcome barriers to maintaining strong Cores; encouraging continuation of growing Cores and building bonds to benefit from strengths around them.

4.3. Stress Management Training. The control training condition will

Stress Management training was 1-hr over of stress response system and impact of stress on health; strategies to manage stress including cognitive and behavioral; and practice of guided relaxation. A 1-hr booster was provided after one month. First hour includes two videos: a summary of stress effects on health and an overview of guided relaxation techniques. Trainers lead interactive discussions. The booster includes a video that summarizes strategies for identifying and changing cognitive distortions that can magnify stress. Participants are invited to sign up to receive informational text messages for six months (approximately one per week). Training was conducted by one of two trainers from University of Rochester.

4.4. Text Messages to Reinforce Training Concepts. Participants in both training conditions will be sent post-training informational and motivational text messages, approximately one message per week for the duration of the study. The messages aim to: increase retention of intervention content over time; reinforce group norms regarding intervention practices and content; and encourage personal reflection. Several messages will contain links to a video. In Wingman-Connect condition videos will include the "Class Legacy video" created during the third training session and links to clips from the 'Four Cores Update' videos that participants may contribute to during the follow-up period. In the Stress Management condition videos will include brief clips on stress response and stress management. A small portion of the messages will "poll" participants, e.g., in the Wingman-Connect condition: "Which of the Four-Cores are you finding most helpful in easing your transition to your new base? Text back 1 for Kinship, 2 for Purpose, 3 for Balance, and 4 for Guidance." Group response will be tallied and sent back to the group to promote norms and group ownership of the concepts. Participation in the polls may be examined as an indicator of engagement with the text messages.

No protected health information (PHI) will be sent via text message in either direction. In the consent form (see attached), participants will be informed that both interventions have text message components.

5. Assessment Measures and Strategy.

5.1. Assessment Approach. The assessment package and procedures were selected to:

a. Assess key outcomes and hypothesized mediators multiple times (baseline, 1-month and 6-month follow-up).
b. Create assessment procedures that are sustainable and will retain participants by minimizing participant burden: computerized adaptive testing that maximizes efficiency and brief, validated measures. Total time to complete assessment is projected at 20-25 minutes on a tablet.
c. Protect Airmen's confidentiality regarding their self-report of mental health, by de-identifying sensitive data before it is received by the study team.
d. Collect high-quality data by using measures of outcomes that have considerable reliability and validity and have been used with active military populations.

Unless noted otherwise, all measures will be administered on three occasions: (1) at baseline immediately following enrollment (before training), (2) at 1-month follow-up (while AiTs are still in technical training), and (3) 6-month follow-up.

5.2. Measures

Demographic Information

At baseline, participants will be asked to provide: age (range 18-32+), sex, race/ethnicity, educational background, and prior

military service (e.g., ROTC). At 6-month follow-up questions will assess base type (i.e., CONUS, OCONUS) and whether base is rural, metropolitan or outside the U.S. (base name or location will not be assessed).

Training Satisfaction. Participants will be administered Foran's (2013) three-item scale to assess satisfaction with training. Questions (e.g., perceived value, ability to use training) are rated on a four-point scale: not at all, a little, somewhat, very much. This measure will be administered only at the 1-month follow-up.

Outcome Measures: Mental Health and Tech Training Outcomes

Suicide Ideation and Depression: Computerized Adaptive Testing-Mental Health (CAT-MH) (Gibbons et al., 2017; Gibbons et al., 2012). To reduce participant burden and promote valid responses to questions about mental health, we will use two sub-domains of the CAT-MH to measure symptoms of suicidal ideation severity (CAT-SS) and depression (CAT-DI). The CAT-MH developer (Robert Gibbons, PhD) is a co-investigator and has worked with the study team to optimize the measure and administration for this RCT of a universal prevention program with a military population.

The CAT-Suicide Scale (CAT-SS) and CAT-Depression Inventory (CAT-DI), are dimensional measures that produce continuous severity scores based on symptomatology experienced in the past two weeks. In contrast to traditional assessment tools in which all items are administered to all subjects, the CAT-MH uses a computerized adaptive approach that begins with a large "bank" of items (1008 items) and adaptively administers (based on a respondent's ongoing answers) a small and statistically optimal subset of the items (on average 12 items for each domain), while maintaining a correlation of close to r=0.95 with the entire bank of items for each test (389 depression items) and sensitivity of 0.95 and specificity of 0.87. Thus, with only 12 items in 2 minutes, the CAT-MH can extract the information contained in hundreds of items in the item bank (Gibbons et.al. 2012).

The CAT-SS item bank draws on 111 statistically related items from depression and anxiety domains that are syndromally associated with suicidality. The CAT-SS also includes 11 items from well-validated scales specific to assessing risk for suicidal ideation or behavior. To encourage valid responses in this military population, the questions that participants will be administered are 'indirect' to suicide (e.g., I felt that life was empty or wondered it was worth living."). We will not administer a single question currently in the CAT-SS item bank that directly queries about self-harm thoughts or behaviors - "Did you think about taking your own life"- in light of evidence that military personnel are reluctant to endorse any level of personal suicide concerns (e.g., Vannoy et al., 2016). Removal of this single item from the 111-item bank will not impact the validity of the adaptive suicide scale scores because there are several other items regarding suicidal ideation with similar or even higher discrimination (Gibbons et al., in press) and similar item threshold.

The CAT-SS yields a continuous score indicating the probability of current suicidal ideation, rather than a discrete score indicating that an individual is suicidal. In this study of a general AF population, the focus will be on testing the impact of the universal intervention on reducing risk across the population (reflected by this continuous score), rather than on high risk individuals. CAT-SS also yields categorical scores (low, medium, high); an increase in category is associated with 16 times greater likelihood of serious suicidal ideation within the next 6 months and increased likelihood of suicidal behavior (Gibbons et al., 2017). We combined medium and high categories for this non-clinical sample. Using the same adaptive technology, CAT-DI yields a dimensional measure of *depression severity* (0-100); when thresholded (>35) CAT-DI scores have high sensitivity and specificity for diagnosis of major depressive disorder.

As an added protection to participants' confidentiality, responses regarding mental health (CAT-DI and CAT-SS) will be completed on a secure server separate from other responses and stored by CAT-MH administrator that has no ability to link those data to specific individuals; identifiers will be removed CAT-MH data before that data is sent to the research team (see below for details).

Occupational Functional Impairment. Participant functioning will be assessed with a set of 5 yes/no questions about behavioral based indicators, used to validate the Walter Reed Functional Impairment Scale (Herrell et al., 2014): if superior has expressed concerns about work performance, if Airman has received corrective training for substandard performance, received a negative counseling statement, received a Letter of Reprimand. Validity of the measure is supported by correlation with independent measures of psychosocial functioning and wellbeing with a military population (Herrell et al., 2014).

Measures of Class/Unit Protective Processes

*Cohesion***.** We will use a 3-item scale used in prior research with active military units (Williams, et al., 2016). The questions ask each participant if members of their class are cooperative with each other, know they can depend on each other and stand up for each other; each question is rated on a four-point scale (Strongly agree to Strongly disagree). Pilot testing during intervention developed showed high reliability of this 3-item scale (Cronbach's alpha of 0.83). The items will be administered at baseline and 1-month follow-up only during technical training phase.

Class Relationship Networks: We will use standard social network tools to assess characteristics of relationship networks among class members (Valente, 2009). Each participant will be asked to name the individuals in in their tech class who "you respect and would choose to spend time with" (up to 5). It is necessary to collect names in order to create 'social networks' that include information about which individuals name each other. Names will not be retained (names deleted and replaced with code numbers). Measures of total network ties, including in-nominations (friend nominations received) and out-nominations (friend nominations made) and reciprocity of nominations, will be calculated for individuals and for the total class. Variables indicative of class cohesion will include density of ties and reciprocity of ties. Nominations will be used at baseline to identify Key Wingman (select participants with greatest number of nominations received and reach to others).

*Morale***.** Participants will be asked to rate their unit (class) morale with a single item on a 5-point scale (1 *very low* to *5 very high*). This measure has also been validated in other studies (Britt & Dickinson, 2006). Class morale will be assessed at baseline and 1-month follow-up. Personal morale will be rated on a 5-point scale (1 *very low* to 5 *very high*). This single-item measure has good validity in military samples (Britt & Dickinson, 2006; Britt, Dickinson, Moore, Castro, & Adler, 2007). A class measure of aggregate morale will be created by combining class ratings at baseline and 1-month follow-up.

Help-Seeking Acceptability (Schmeelk-Cone, Pisani, Petrova, & Wyman, 2012; Wyman et al., 2010). This scale assesses perceptions of friends' and family members' support for getting help (perceived norms), intentions to seek help for distress, and expectations of receiving help. Respondents rate each item using a four-point Likert scale (Cronbach's α=0.86–.90). HSA has been linked with greater likelihood that distressed adolescents sought help (Pisani et al., 2012).

Healthy Class Norms and Practices: To assess perceptions that classmates support behaviors that strengthen protective factors taught through Wingman-Connect training, we developed and piloted a 6-item scale. Each items begins with a stem "Students in my tech class…": 'take steps to learn about each other's strengths', 'work towards a healthy balance between studying and healthy activities', 'pay attention to growing career goals and direction', pay attention to growing career goals and direction', learn from each other how to be a better airman', would encourage me to seek help for an issue before it becomes a problem'. These will be rated on a four-point scale from Strongly Agree to Strongly Disagree. These items will be used at baseline and 1-month follow-up. During piloting these yielded a scale with Cronbach's alpha of .87.

Measures of Individual Risk and Protective Factors

Loneliness-Social Disconnection. A three-item version of the UCLA Loneliness Scale (Hughes, Waite, Hawkley, & Cacioppo, 2004) will be administered to assess extent to which participants' feel they lack companionship, feel left out and feel isolated from others (Hardly Ever, Some of the Time, Often). These items will be administered at all three assessment points.

Thwarted Belongingness and Perceived Burdensomeness. We will use items from the Interpersonal Needs Questionnaire (Van Orden, K. A., Cukrowicz, K. C., Witte, T. K., & Joiner, T. E., 2012) to measure thwarted belongingness and perceived burdensomeness on a seven-point scale, Not at all true for me (0) to Very true for me (6), and will be administered at all three assessment points.

Difficulties with Emotion Regulation (DERS) (Gratz & Roemer, 2004). The 8-item subscale, Limited Access to Strategies for Emotion Regulation, will be administered to assess the degree to which an individual feels powerless to manage emotional reactions and recover from feelings of upset. This scale has high reliability and evidence of validity based on self-report of behavioral and emotional difficulties.

Maladaptive Coping Attitudes (Gould et al., 2004). This four-item scale (Cronbach's α > .90 in previous studies) evaluates coping attitudes and practices. The four-item scale includes questions about the acceptability of getting help for problems ("People should be able to handle their own problems without outside help."), endorsement of drugs and alcohol as ways to solve problems, and endorsement of suicide ("Suicide is a possible solution to problems"). The scale has been used in numerous studies and higher endorsement of overall maladaptive norms on this scale differentiated adolescents who had made suicide attempts from those who had not (e.g., (Gould et al., 2004; Schmeelk-Cone et al., 2012; Wyman et al., 2010).

Behaviors for Career Protective Factors. To assess behaviors to strengthen protective factors, we developed six items inquiring about the frequency in the past month. Each item begins with a stem "In the past month how often have you: sought out instructors or MTLs to get mentoring?" Taken steps to strengthen a healthy relationship? Drawn strength or support from

a relationship to overcome a challenge? Dedicated time to a healthy activity to stay balanced? Taken steps to further my purpose and career goals? Items are rated on a four-point scale: never, a few times, several times, almost every day.

Alcohol Use Disorders Identification Test-C (AUDIT-c) (Bush et al., 1998). The AUDIT-C is a 3-item scale designed to identify persons who are hazardous drinkers or have active alcohol use disorders (including alcohol abuse or dependence), based on a modification of the 10-item AUDIT. The AUDIT-C can be used as a continuous indicator of potential problem drinking. Research shows acceptability sensitivity and specificity including for use with veteran populations (Bradley et al., 2003).

Sleep Problems-Insomnia Severity Index (Bastien et al., 2002). Sleep problems will be assessed with the four-item version of the Insomnia Severity Index (ISI; Bastien et al, 2001; Adler et al., 2009). Two items will rate the current severity of difficulty falling asleep and staying asleep (scale: None, Mild, Moderate, Severe, Very Severe). One asks how satisfied/dissatisfied the respondent is with their current sleep pattern (scale: Very satisfied, Satisfied, Neutral, Dissatisfied, Very dissatisfied). And the last asks about how much sleep problems interfere with daily functioning (scale: Not at all/No sleep problem, A little, Somewhat, Much, Very much interfering).

Dimensions of Anger Reactions-5 (Forbes et al., 2014). Anger will be assessed by the DAR-5 (Forbes, et al., 2014), a five-item scale. Specific items inquire about getting angry, staying angry, intensity of anger and anger affecting getting along with others (response scale: None or almost none of the time, A little of the time, Some of the time, Most of the time, All or almost all of the time). The DAR-5 yields a continuous sum scores that has strong internal reliability and concurrent validity with longer measures of trait anger.

Social and Personal Military Functional Impairment. Participant functioning will be assessed with two subscales from the Walter Reed Functional Impairment Scale (Herrell et al., 2014): Social and Personal. Social functioning is a 4-item scale on difficulty getting along with coworkers, interacting with social groups, getting along with family or friends or ability to have a close relationship. Personal functioning has two items on difficulty handling personal responsibilities and getting bills paid on time. Both are rated on a five-point scale: No difficulty at all, a little difficulty, moderate difficulty, quite a bit of difficulty, extreme difficulty. Validity of the measure is supported by correlation with independent measures of psychosocial functioning and wellbeing with a military population (Herrell et al., 2014).

Technical School Training Completion. Each participant's AFSC qualification information at the end of technical training (i.e., graduated, 'washed back' for retraining, or separated from the AF) will be collected from the squadron administrator directly into REDCap on the University of Rochester secure platform. We will also collect information pertaining to subject participation in other prevention training programs offered to technical training classes. This information will be merged with self-report data (and de-identified before being sent to the research team, at the same time other data are de-identified. See below). This information will be used to determine if Wingman-Connect increases successful and timely completion of technical training and qualification to perform duty (AFSC being trained for).

6. Data Collection, Storage and Confidentiality.

6.1. **On-Line Assessment Process, Storage, De-Identification**.

Assessments will be conducted using an approach that we developed through our prior NIMH-funded randomized trials of Sources of Strength with 40 secondary schools involving more than 15,000 participants. This on-line process has several advantages for protecting safety and confidentiality of subjects in a population-level prevention trial such as this one.

Enrolled subjects will be given an access code to enter a secure server on the web-based University of Rochester REDCap System using a hand-held tablet. REDCap servers are housed in a secure, local data center at the University of Rochester administered by the Department of Bioinformatics, and all web-based information transmission is encrypted. REDCap was developed in a manner consistent with HIPAA security requirements and is recommended to University of Rochester researchers by the URMC Research Privacy Officer and Office for Human Subject Protection. Security for the database is managed in accordance with industry standards for Role Based Access Control (RBAC) to create custom roles and permission for the project and assign roles to each user (e.g., administrator). Passwords are one-time use and will be managed in accordance with URMCs institutional policies. At baseline and at the 1-month follow-up assessments, University of Rochester research personnel will be present for the assessments, which will be held in the same private location as enrollment (with no leadership present); research personnel will ensure that participants have adequate space, privacy and opportunity to ask questions. At the 1 month follow-up, we will ask participants to confirm their primary and backup contact information so that we may contact them for the 6-month survey and participant payment.

For the 6-month follow-up assessment, participants will be sent a URL to the email address or cell phone number provided at enrollment (on the Consent form). The URL will provide a single-time use access to the REDCap system. If participants do not complete the survey, we will send two reminders through email (or phone call). Emails will be sent from a dedicated, password protected email account behind University of Rochester firewalls, so that email addresses provided by participants are protected and can be deleted once surveys are complete.

During the on-line assessment, participants in the REDCap platform will be directed to the CAT-MH™ through an embedded URL to answer questions about behavioral and emotional health. CAT-MH is a separate, secure web application that administers a series of questions (i.e., "interview") using computer adaptive testing algorithms to maximize efficiency. The component runs on a Java application server and reside on a U.S.-located, hardened server and store results in a SQL Server database. The client interface is based on HTML5 and all communication between it and the server use an encrypted transport layer (256-bit SSL encryption) for privacy and message integrity. Besides the standard server practice of logging Internet Protocol (IP) addresses for auditing purposes, the only information collected as part of the interview process is the start time of the interview, the questions adaptively administered, the subject's response to each question and the length of time to select that response. The questions asked and the responses selected during the interview are encoded based on table indexes in a separately encrypted and secured database. The databases are only accessed by the web applications and are not 'exposed' to the Internet at all. No data are stored on the participant. The only information related to an individual in the database is an encoded identifier to uniquely identify her within the context of the database. At the end of the project the CAT-MH administrator will send data through a secure, encrypted VPN to the URMC RED-Cap administrator for the study.

De-Identification of Data. Individual data on REDCap are encrypted and identified only by an ID. **The Department of Bioinformatics will not have access to any participant names or other identifiers**, which provides an additional source of protection for confidentiality of the data. The ID numbers used to log onto the website (and the ID assigned on the CAT-MH records) will be changed by the URMC Department of Bioinformatics to a new assigned ID before any survey data is sent to Dr. Wyman's research team or to Northwestern University for data analyses. Thus, the data are de-identified, although each individual's data is linked over time through the new assigned ID. Phone numbers for text messaging and email addresses for reminders and the final survey will be kept separate from the survey data.

The only other potential identifiers are audio recordings and public service announcements. Neither will be retained as data, and in no way can subjects' data be linked to those recordings/images.

6.2. Data from Nominations of Key Wingmen.

Participants will be asked to nominate classmates whom they respect and would choose to spend time with (described in Social Network measures above). These nominations will be made on a REDCap survey separate from questions about fitness and health, so the study team can access nominations to identify well-connected Key Wingman without accessing responses on fitness and health (which are de-identified before being sent to the research team). Likewise, subjects will name instructors and MTLs using social network measures (names are required). Names of classmates and instructors/MTLs will be changed to ID numbers within the database at the earliest convenience and names deleted. Any names of other people will either be given numbers (such as instructors) or given designations (such as 'friend', 'teacher', etc.).

6.3. Audio Recording of Training.

Trainings will be audio recorded for quality control purposes only and not retained as study data. Recordings will be made directly onto a secure, encrypted hard drive, and uploaded to University of Rochester secure servers. Participants will be informed that trainings will be audio taped. A URMC research staff member will review to 'check off' that all training components were delivered, and then the audio recordings will be deleted at the earliest opportunity.

6.4. Text Message System, Email, and Video Storage.

To distribute text messages, we have selected a commercial SMS service that meets evolving healthcare industry standards, including compliance with HIPAA, HITECH, HITRUST and ISO. In order to personalize text messages (which was requested by pilot participants and is suggested as best practice in the literature, Fjeldsoe, Marshall, & Miller, 2009; Head et al., 2013), participants' last names will be entered into the texting system along with their phone number. No sensitive information will be sent or received and stored. As noted in Text message description above, a few "polls" will be conducted that invite optional 'numerical" responses (e.g., choose 1, 2, or 3 indicating which 'Core' you are prioritizing to strengthen). Participation in polls and clicks onto the videos may be examined as indicators of involvement with the text messaging. Participants will be informed that texts are computer-generated and are not for texting interaction with study personnel. Participants will be informed how they can stop receiving texts at any time.

Emails for the follow-up survey at 6-months will be sent from a dedicated, password protected email account behind University of Rochester firewalls, so that email addresses provided by participants are protected and can be deleted once surveys are complete.

Public service campaign videos in Wingman-Connect will be recorded during training on phones or cameras and stored in encrypted hard-drives. The second campaign, the Four Core Updates, participants are contacted by texting (they provide cell phone number when they sign up to participate in those videos) and asked to provide a 20-min time-slot during off-duty hours, and choose a preferred video call platform (skype, google hangout, Face Time). After reminding the participant that the chat will be recorded for "Four core Update video", the chat is recorded using ScreenFlow and stored directly on an encrypted hard-drive. Edited videos will be stored on Wistia.com and can be accessed via a private URL that is accessible within login but not indexed on the web (i.e., cannot be found via search engine). Participants will be informed about how videos are stored, and their participation is voluntary. No videos will contain sensitive information and will not be retained as study data.

7. Risk/Benefits and Measures to Minimize Risks and Safety Monitoring

7.1 Risks to Subjects. (1) During training in both Wingman-Connect and Stress management conditions, some of the topics discussed include common stressors and challenges, which could elicit stress and/or distress; (2) Subjects could disclose personal information - such as what was challenging in adjusting to AF life and impact on family relationships - and another participating subject could repeat that information out of the training context, resulting in a loss of confidentiality. (3) The text messages and emails that are sent to participants as part of the intervention (optional) or for follow-up assessment (required) contain no personal information, but these messages could be accidently seen by another person who has physical access to their phones or computers, revealing the subject's participation in this training study. (4) In completing questionnaires about emotional distress (e.g., depression, hopelessness), participants may become aware that they, or someone else, requires help (discovery).

7.2 Measures to Minimize Risks.

(1) Each training will be facilitated by one or more senior trainers (lead trainer Ms. DeVelder, Mr. Yates, Dr. Pisani, Dr. Wyman). All have extensive experience in leading trainings and have attended Sources of Strength training for trainers. The facilitators will pay close attention to group process, including indications that any subject is distressed or indicating need for further follow-up. At the beginning and end of each group, the facilitators will invite members of the group to contact them to discuss any concerns about their experiences in the group. The facilitators will have information on each site about behavioral health resources and can provide information about resources for help to any subject who requests this information.

(2) To protect privacy and confidentiality, subjects will be reminded at the beginning and end of each session that content shared by individuals should not leave the group. During the consent process (and in the consent document), subjects will be notified that their privacy and confidentiality cannot be guaranteed and they should be mindful when sharing personal experiences.

(3) Participants will be reminded that other people who have physical access to their phones or computers could see the texts and emails that they receive as part of this study; the consent form will advise subjects to use secret passcodes if they wish to keep their participation in this training study private.

(4) **All Participants Informed About Mental Health Resources**. All participants completing the assessment will receive information about mental health resources should they require help for themselves or for another individual (information will appear a screen during the assessment). As in our prior RCTs with Sources of Strength with secondary school populations, the project team has determined that greatest population benefit comes from providing resource information to all participants in this type of RCT testing a universal prevention program (not a clinical population). In order to address reluctance that many participants may experience in seeking help from mental health services on base, the information will include the number for the National Suicide Prevention Lifeline. Emerging data suggests that young adults such as this technical training population are more likely to access on-line services or chat rooms versus telephone call-in numbers.

7.3 Benefits to Subjects. Subjects could benefit by learning strategies for strong relationships and managing stress and challenges of Air Force life. This information could help them to apply those strategies in their own lives to improve resilience and relationships.

8. Data Analysis and Data Monitoring

8.1 Specific Analyses for Phase II Randomized Trial

After receiving de-identified data, analyses will be completed by Dr. C. Hendricks Brown, Director of the Center for Prevention Implementation Methods (Northwestern University), working closely with the URMC Project Director and Team.

For hypotheses that Wingman-Connect will reduce risk for suicide ideation and individual symptoms of depression, as well as self-reported problems with technical training course, we will use multilevel growth models in Mplus (Brown, Costigan, & Kendziora, 2008; Muthén & Asparouhov, 2003; B. Muthén, 2004; Muthén & Muthén, 2012). This modeling will allow us to examine the change in these variables over time. In particular, at the individual level, we will model subject i's response on pertinent measures at time t (baseline, 1 month, 6 months) in the gth workforce group, Y_{igt}, as having a linear growth model that is specified by a random intercept a_{ig} and random slope b_{ig}, Y_{igt},= a_{ig}+ b_{ig} t + ε_{igt} where the last term is an independent error representing variation of each observation about one's linear trajectory. In this model, the slopes b_{ig} carry the information about intervention effect, and because randomization is at the level of the group, the overall change in symptoms and behaviors due to Wingman-Connect ($H_g = 1$) compared to control ($H_g = 0$), is modeled as $b_{ig} = \mu + \beta H_g + \gamma_g + \varepsilon_{ig}$. Here β is the mean difference in slopes for Wingman-Connect versus control, and the two error terms, γ_g and ε_{ig}, represent variation across and within groups respectively. The test of change will be based on a Wald-type test of the magnitude of the estimate of β relative to its standard error. Both standard fit indices for latent variable models and diagnostics (C. Wang, Hendricks Brown, & Bandeen-Roche, 2005) will be used to examine the fit and if necessary improve the model. These analyses are similar to those we have reported before for antidepressant effects on depressive symptoms (Gibbons, Hur, Brown, Davis, & Mann, 2012) and suicide related outcomes (Gibbons, Brown, Hur, Davis, & Mann, 2012). We will apply the same analytic approach to test the hypothesis that Wingman-Connect will increase healthier social connectedness, unit integration, and norms for coping relationships and transitions,

We will examine the mediational influence of social connectedness and emotion regulation on depression and suicide risk symptoms by testing whether the product of the two relationships, the first from intervention condition to connectedness/emotion regulation, and the second from mediators to symptoms, is significantly different from zero (MacKinnon & Luecken, 2008). These analyses will also examine the interactive effect of intervention by proposed mediator (Gibbons et al., 2012).

We also plan to conduct additional mediation analyses that clarify how the putative intervention effect is being achieved. Specifically, we hypothesize that Wingman-Connect would work when more healthy relationship ties are created and strengthened among those in the same workforce group. These analytic models will allow us to assess how the level of implementation of Wingman-Connect, including its reach to those airmen who are originally on the periphery of the social networks, receive messages and find them useful.

In all of these analyses, we will handle missing data using full information likelihood methods (FIML), which is known to reduce bias and outperform other procedures such as throwing away incomplete cases or using last observation carried forward (Siddique et al., 2008) To reduce the chances of finding spurious results due to the large numbers of statistical tests, we are relying mostly on growth models rather than repeated analyses at single time points. Also, we plan to incorporate similar measures (e.g., suicide risk and depression) in the same growth model. As a third approach to reducing the chance of spurious findings, we will conduct any tests for model development, e.g., those that would check for nonlinearity, for example, by blinding the decision-making for arriving at a best-fitting model to any potential effects due to intervention. That is, all tests of intervention will be done after identifying best fitting models (Kellam et al., 2008).

Statistical Power. Using Optimal Design (OD) software, we computed the effect size that we expect to obtain in this group-based randomized trial on detecting a change in the continuous measure of suicide risk (CAT-SS). We assumed there would 160 groups with a total of 1550 airmen, and over a 6-month period the attrition rate would be 25%. We set a Type I error rate of 5%, power at 80% and assumed a small intra-class correlation ranging from 0.02 to 0.05 on the primary outcome of interest, the suicide scale predictor. Conservatively, we can achieve 80% power in this design with an effect size of 0.18 when the ICC is 0.02 and an effect size of 0.20 when the ICC is 0.05.

7.4. Data Safety Monitoring. A Data Safety Monitoring Committee (DSMC) will be comprised of: (a) Col Steven Pflanz, MD; US-AF Director of Psychological Health; (b) Robert Klesges, PhD., Professor, University of Tennessee Health Sciences Center. Dr. Klesges currently has four federally funded projects testing alcohol, smoking and other prevention programs with the US-AF. (c) Wilfred Pigeon, PhD, Director of the Veterans Affairs Center for Excellence in Suicide Prevention and Associate Professor of Psychiatry, University of Rochester Medical Center. The PI (Dr. Wyman), co-investigators (Pisani, Brown) and Senior Project Coordinator (Mr. Yates), while not members of the DSMC, will be available for each to meeting to provide a progress report of the study, answer questions and receive additional guidance from the DSMC, including requests for additional information.

The DSMC will meet every six months during the course of the RCT to review interim data, and may schedule additional meetings as needed. In advance of those meetings the members will receive data summarizing intervention (Wingman-Connect) and control (Stress Management Training) groups. Data selected for interim reports will include comparison of the intervention and control groups on: continuous measures of depression and suicide risk scales (CAT-DD and CAT-SI), technical school graduation/completion; functional impairment; loneliness/social disconnection. Obtaining data for DSMC review will follow the study protocol of de-identification prior to being sent to the study team. A major aim of this study is to determine if Wingman-Connect compared to Stress Management training reduces risk for suicidal ideation using the continuous measure of risk (CAT-SS). Along with interim data summary, the chief statistician (Dr. C. Hendricks Brown) will oversee analyses computing whether suicide ideation risk varies by training condition (at each DSM report).

The DSMC will be informed of any unanticipated problems that may occur during recruitment or participation in training or assessments. The DSMC will also be informed of any adverse advents that may occur (e.g., deaths) and come to the attention of the project team, including those unrelated to study participation. The study team is likely to be aware of adverse events during the 2-4 months that participants are at Sheppard Air Force Base for Technical training, but is unlikely to be aware of deaths or other serious adverse events that occur after participants have transferred to operational air force bases.

References

Adler, A. (2016, personal communication). Personal Integration scale.

Adler AB, Bliese PD, McGurk D, Hoge CW, Castro CA. (2009). Battlemind debriefing and battlemind training as early interventions with soldiers returning from Iraq: randomization by platoon. J Consult Clin Psych, 77: 928–40.

Bastien, C. H., Vallieres, A., & Morin, C. M. (2001). Validation of the Insomnia Severity Index as an outcome measure for insomnia research. Sleep Medicine, 2, 297-307.

Britt, T.W. & Dickinson, J.M. (2006). Morale during military operations: A positive psychology approach. In Britt, Castrol, Adler (Eds). *Military life: The psychology of serving in peace and combat: Military performance., Vol. 1 (pp. 157-184). xi, 255 pp. Westport, CT: Praeger Security International.*

Britt, T.W., Dickinson, J.M., Moore, D., Castro,C.A. & Adler, A. (2007). Correlates and consequences of morale versus depression under stressful conditions. *Journal of Occupational Health Psychology, 12(1),* 34-47.

Brown, C.H., Costigan, T.E. & Kendziora, K.T. (2008). *Data analytic frameworks: Analysis of variance, latent growth, and hierarchial models.* In Nezu,A.M. & Nezu, C.M. (Eds). Evidence-based outcome research: A practical guide to conducting randomized controlled trials for psychosocial interventions (pp. 285-313). New York, NY: Oxford University Press.

Brown, C.H., Ten Have, T.R., Jo, B., Dagne, G., Wyman, P.A., Muthén, B., Gibbons, R.D. (2009). Adaptive designs for randomized trials in public health. *Annual Review of Public Health*, **30**: 1-25.

Brown, C.H., Wyman, P.A., Guo, J. & Pena, J. (2006). Dynamic wait-listed designs for randomized trials: New designs for prevention of youth suicide. *Clinical Trials*, **3**(3): 259-71.

Bush, K., Kivlahan, D. R., McDonell, M. S., Fihn, S. D. and Bradley, K. A. (1998) The AUDIT Alcohol Consumption Questions (AUDIT-C): an effective brief screening test for problem drinking. *Archives of Internal Medicine,* 158, 1789–1795.

Department of Defense. (2012). *Department of defense suicide event report (DoDSER)*

Foran, H.M., Garber, B.G., Zamorski, M.A., Wray, M., Mulligan, K., Greenberg, N., Castro, C.A. & Adler, A. (2013). Postdeployment military mental health training: Cross-national evaluations. *Psychological Services*, 10(2): 152-160.

Forbes, D., Alkemade, N., Mitchell, D., Elhai, J.D., McHugh, T., Bates, G., Novaco, R.W., Bryant, R. & Lewis, V. (2014). Utility of the dimension of anger reactions-5 (DAR-5) scale as a brief anger measure. *Depression and Anxiety*, 31, 166-173.

Gibbons, R.D., Hur, K., Brown,C.H., Davis, J.M. & Mann, J.J. (2012). Benefits from antidepressants: synthesis of 6-week patient-level outcomes from double-blind placebo-controlled randomized trials of fluoxetine and venlafaxine. *Archives of General Psychiatry, 69*(6), 572-9.

Gibbons, R.D., Brown, C.H., Hur, K., Davis, J.M. & Mann, J.J. (2012). Suicidal thoughts and behavior with antidepressant treatment: reanalysis of the randomized placebo-controlled studies of fluoxetine and venlafaxine. *Archives of General Psychiatry, 69*(6), 580-7.

Gibbons, R.D., Kupfer, D., Frank, E., Moore, T., Beiser, D.G., & Boudreaux, E. (2017). Development of a computerized adaptive suicide scale: CAT-SS. *Journal of Clinical Psychiatry*, in press.

Gibbons, R.D., Weiss, D. J., Pildonis P., et al. (2012). Development of a computerized adaptive test for depression. *Archives of General Psychiatry*, 69(11): 1104 – 1112.

Gould, M. S., Velting, D., Kleinman, M., Lucas, C., Thomas, J.., & Chung, M. (2004). Teenagers' attitudes about coping strategies and help-seeking behavior for suicidality. *Journal of the American Academy of Child & Adolescent Psychiatry, 43*(9), 1124-1133.

Gratz, K.L., & Roemer, L. (2004). Multidimensional assessment of emotion regulation and dysregulation: Development, factor structure, and initial validation of the difficulties in emotion regulation scale. *Journal of Psychopathology and Behavioral Assessment, 26*(1), 41-54.

Herrell, R.K., Edens, E. N., Riviere, L.A., Thomas, J.L., Bliese, P.D. & Hoge, C.W. (2014). Assessing functional impairment in a working military population: The Walter Reed functional impairment scale. *Psychological Services*, 11(3): 254-264.

Hoge, C.W., Terhakopian, A., Castro, C.A., Messer, S.C. & Engel, C.C. (2007). Association of posttraumatic stress disorder with somatic symptoms, health care visits, and absenteeism among Iraq war veterans. *American Journal of Psychiatry, 164*, 150-153.

Hughes, M.E., Waite, L.J., Hawkley, L.C. & Cacioppo, J.T. (2004) A short scale for measuring loneliness in large surveys: Results from two population-based studies. *Research on Aging, 26(6),* 655-672.

Institute of Medicine Committee on the Assessment of Readjustment Needs of Military Personnel, Veterans, and their Families. (2013). *Returning home from Iraq and Afghanistan: Assessment of readjustment needs of veterans, service members, and their families.* Washington, DC: The National Academies Press Washington, DC.

Kellam S.G., Brown, C.H., Poduska, J.M., Ialongo, N.S., Wang, W., Toyinbo, P., Petras, H., Ford, C., Windham, A. & Wilcox, H.C. (2008). Effects of a universal classroom behavior management program in first and second grades on young adult behavioral, psychiatric, and social outcomes. *Drug and Alcohol Dependence, 95 (Suppl 1), S5-S28.*

LoMurray, M. (2005). *Sources of strength facilitators guide: Suicide prevention peer gatekeeper training*. Bismarck, ND: The North Dakota Suicide Prevention Project.

MacKinnon, D.P. & Luecken, L.J. (2008). How and for whom? Mediation and moderation in health psychology. *Health Psychology, 27*(2, Suppl), S99-S100.

Muthén, B. O., & Asparouhov, T. (2003). Advances in latent variable modeling, Part I: Integrating multilevel and structural equation modeling using Mplus. Unpublished manuscript.

Muthén, B. (2004). Latent variable analysis: Growth mixture modeling and related techniques for longitudinal data. In D. Kaplan (ed.), Handbook of quantitative methodology for the social sciences (pp. 345-368). Newbury Park, CA: Sage Publications.

Muthén, L.K., & Muthén, B.O. (2012) Mplus: Statistical analysis with latent variables: User's Guide (5th ed.). Los Angeles:Authors.

Petrova, M., Wyman, P.A., Schmeelk-Cone, K. & Pisani, A. R. (2015). Positive-themed suicide prevention messages delivered by adolescent peer leaders: Proximal impact on classmates' coping attitudes and perceptions of adult support. *Suicide and Life Threatening Behavior*, *45*(6): 651-663

Pisani, A. R., Schmeelk-Cone, K., Gunzler, D., Petrova, M., Goldston, D. B., Tu, X., & Wyman, P. A. (2012). Associations between suicidal high school students' help-seeking and their attitudes and perceptions of social environment. *Journal of Youth and Adolescence, 41*(10), 1312–1324

Pisani, A. R., Wyman P.A., Mohr, D.C., Perrino,T., Gallo,C., Villamar, J., Kendziora, K., Howe, G.W., Sloboda, Z. & Brown, C.H. (2016). Human subjects protection and technology in prevention science: Selected opportunities and challenges. *Prevention Science, 17(6),* 765-778.

Ritchie, E. C., Keppler, W. C., & Rothberg, J. M. (2003). Suicidal admissions in the United States military. *Military Medicine, 168*(3), 177-181.

Russell, D. W. (1996). UCLA Loneliness Scale (Version 3): Reliability, Validity, and Factor Structure. *Journal Of Personality Assessment*, *66*(1), 20.

Schmeelk-Cone, K., Pisani, A., Petrova, M. & Wyman, P.A. (2012). Three scales assessing high school students' attitudes and perceived norms about seeking adult help for distress and suicide concerns. *Suicide and Life Threatening Behavior, 42*(2):157-72.

Siddique J, Brown, C.H., Hedeker, D, Duan, N., Gibbons, R.D., Miranda, J. & Lavori, P.W. (2008). Missing data in longitudinal trials – Part B, Analytic issues. *Psychiatry Annals*, *38*(12), 793-801.

Valente, T.W., Fujimoto, K., Chou, C.P. & Sprijt-Metz, D. (2009). Adolescent affiliations and adiposity: A social network analysis of friendships and obesity. *Journal of Adolescent Health, 45*(2), 202-4.

Vannoy, S.D., Andrews, B.K., Atkins, D.C., Dondanville, K.A., Young-McCaughan, S. & Peterson, A.L. (2016). Under reporting of suicide ideation in US Army population screening: An ongoing challenge. *Suicide and Life-Threatening Behavior.*

Wang C. P., Brown, C. H., and Bandeen-Roche K (2005). Residual diagnostics for growth mixture models: Examining the impact of a preventive intervention on multiple trajectories of aggressive behavior. *Journal of the American Statistical Association*, 100, 1054-1076.

Williams, G. C., Freedman, Z.R., & Deci, E. L. (1998). Supporting autonomy to motivate glucose control in patients with diabetes. *Diabetes Care, 21,* 1644-1651.

Williams, J., Brown, J. M., Bray, R. M., Anderson Goodell, E. M., Rae Olmsted, K., & Adler, A. B. (2016, March 17). Unit Cohesion, Resilience, and Mental Health of Soldiers in Basic Combat Training. Military Psychology. Advance online publication. http://dx.doi.org/10.1037/mil0000120

Wyman, P. A., Brown, C. H., LoMurray, M., Schmeelk-Cone, K., Petrova, M., Yu, Q., Tu, X., Walsh, E., & Wang, W. (2010). An outcome evaluation of the Sources of Strength Suicide Prevention Program delivered by adolescent peer leaders in high schools. *American Journal of Public Health,* 100(9), 1653-1661.
