# Supplementary material for: Effect of the Wingman-Connect Upstream Suicide Prevention Program for Air Force Personnel in Training: A Cluster Randomized Clinical Trial
Source: JAMA Netw Open. 2020 Oct 21;3(10):e2022532. doi: 10.1001/jamanetworkopen.2020.22532 (PMC7578767; doi:10.1001/jamanetworkopen.2020.22532)
Supplement: Supplement 2. — eAppendix 1. Selection and Matching of Technical Training Classes eTable 1. Matched Pairing of Enrolled Technical Training Classes eAppendix 2. Procedures for Rating Adherence to Wingman-Connect Training Manual eAppendix 3. Modification of CAT-SS Modification of CAT-SS Item Bank by Removing 1 Item eAppendix 4. Protocol Deviations in Statistical Analyses eAppendix 5. Statistical Analyses eTable 2. Baseline Outcomes and Risk/Protective Factors for Full Sample, Wingman-Connect Group and Stress Management Group eFigure 1. Wingman-Connect Impact on Occupational Impairment Indicators in Technical Training eFigure 2. Wingman-Connect Impact on Suicide Severity and Depression Mediated Through Stronger Class Units eFigure 3. P-P Plot of Tests of Baseline Equivalence on Behavior Measures eReferences. [file jamanetwopen-e2022532-s002.pdf]

Supplemental Online Content

Wyman PA, Pisani AR, Brown CH, et al. Effect of the Wingman-Connect upstream suicide prevention program for Air Force personnel in training: a cluster randomized clinical trial. *JAMA Netw Open*. 2020;3(10):e2022532. doi:10.1001/jamanetworkopen.2020.22532

eAppendix 1. Selection and Matching of Technical Training Classes
eTable 1. Matched Pairing of Enrolled Technical Training Classes
eAppendix 2. Procedures for Rating Adherence to Wingman-Connect Training Manual
eAppendix 3. Modification of CAT-SS Modification of CAT-SS Item Bank by Removing 1 Item
eAppendix 4. Protocol Deviations in Statistical Analyses
eAppendix 5. Statistical Analyses
eTable 2. Baseline Outcomes and Risk/Protective Factors for Full Sample, Wingman-Connect Group and Stress Management Group
eFigure 1. Wingman-Connect Impact on Occupational Impairment Indicators in Technical Training
eFigure 2. Wingman-Connect Impact on Suicide Severity and Depression Mediated Through Stronger Class Units
eFigure 3. P-P Plot of Tests of Baseline Equivalence on Behavior Measures
eReferences

This supplemental material has been provided by the authors to give readers additional information about their work.

© 2020 Wyman PA et al. *JAMA Network Open.*

eAppendix 1. Selection and Matching of Technical Training Classes

Eligible classes were those that fit a window of instructional length (37 – 91 days) long enough for participants to complete the 1-month follow-up assessment while still enrolled in their training program, and short enough so that participation in a final 6-month follow-up assessment would occur 2-4 months after transfer to first duty base. Of 1049 classes formed during the enrollment period, 281 were outside the instructional length window; 371 classes were excluded because the class midpoint occurred after one of 26 study enrollment dates. Within each squadron, eligible classes were paired on job classification (AFSC), size, class duration and start date; 181 classes had no suitable pair and were thus excluded. For each of 26 enrollment dates, 4 – 6 class pairs were formed (50 - 70 AiTs), and one class from each of the best-matched pairs was randomly assigned to Wingman-Connect and the other to stress management training. eTable1 below summarizes criteria for matching classes and number of pairings in each category.

Of the 1897 Airmen-in-training in the 216 selected classes, 1732 were exposed to their class' assigned intervention. Those not exposed were 76 Airmen undergoing follow-on retraining (no longer First Term Airmen) and 89 AiTs who chose not to attend or were absent due to other responsibilities.

eTable 1. Matched Pairing of Enrolled Technical Training Classes

**182**	(84.7%)	Category 1	Identical courses with start date <21 days apart
**10**	(4.7%)	Category 2	Identical courses with start date 21-35 days apart
**12**	(5.6%)	Category 3	Similar AFSC course, >80% similar in class size and duration, & with start date <21 days apart
**6**	(2.8%)	Category 4	Similar AFSC course, >80% similar in class size and duration, & with start date 21-35 days apart
**2**	(0.9%)	Category 5	Dissimilar AFSC course, >80% similar in class size and duration, & with start date <21 days apart
**3**	(1.4%)	Anomalous	3 Classes were without pair due to enrollment drop or SQN replacement

Enrollment Capture of Sample Population, 363/365 TRS, October 2017 – January 2019

**20.5%**	(215/1049)	of ALL Technical Training classes in participated in the study
**28.0%**	(215/768)	of classes that met inclusion criteria of 37-91 days duration participated in the study
**54.2%**	(215/397)	of classes that met inclusion criteria, and aligned with an enrollment date participated in the study
**66.4%**	(215/324)	of classes that fit an enrollment and were able to be paired IAW protocol participated in the study

© 2020 Wyman PA et al. *JAMA Network Open.*

eAppendix 2. Procedures for Rating Adherence to Wingman-Connect Training Manual

Wingman-Connect Training Adherence

All Wingman-Connect training sessions were audio recorded directly onto a secure, encrypted hard drive and uploaded to University of Rochester secure servers. To measure trainer fidelity over the course of the study, we adapted methods we previously tested for measuring adherence to a suicide prevention training program that is rolled out over multiple cohorts.[1] We selected, and developed adherence measures for, eight key modules that reflected core elements of the Wingman-Connect program and capture a range of trainer behaviors (e.g., engaging Airmen, clarifying concepts, and eliciting peer-to-peer sharing). Usable audio recordings were available for 95% of selected modules from all 26 training cohorts in the study. Four trainer coders, URMC research staff, rated recordings from all 26 training sessions; all sessions were rated by at least two coders (98.1% inter-rater agreement). Trainer fidelity was high: 97.7%, and we saw no evidence of adherence drift (losing fidelity over time) through the course of the study.

eAppendix 3. Modification of CAT-SS Item Bank by Removing 1 Item

Our Air Force advisory panel and the research team agreed to remove one sensitive question out of 11 that queries about self-harm ("Did you think about taking your own life?"). It was anticipated that Airmen would be reticent to self-identify about direct self-harm intentions due to concerns about impact on their careers,[2] even with assurances that data would be de-identified and not shared with the Air Force. From psychometric considerations, removal of this single item from the 111-item bank would not impact validity of the adaptive suicide scale scores because other items have similar or even higher discrimination.[3]

eAppendix 4. Protocol Deviations in Statistical Analyses

The original statistical analysis plan was modified in the following ways:

1) We originally proposed using growth models to test intervention effects on the primary outcomes from baseline spanning 3-month and 6-month follow-up points. However, because Air Force leadership selected technical training as the intervention site, the length of first follow-up was reduced to 1-month so that it coincided with ending of that training phase. With only 3 time points, growth models estimated for the primary outcomes with the modified follow-up phases were poorly fitting because of different measurement error variances by condition, so we shifted to examining effects from baseline to each follow-up point separately.

2) We originally proposed assessing alcohol use and sleep problems and including those measures as secondary outcomes, because Wingman-Connect was expected to include messaging components that would encourage Airmen to follow healthy substance and sleep behaviors. However, the final Wingman-Connect intervention, developed through Airmen input about priorities, did not include those components. Thus, those measures were not included as secondary outcomes.

© 2020 Wyman PA et al. *JAMA Network Open.*

eAppendix 5. Statistical Analyses

Statistical Analysis

Using Optimal Design (OD) software, we computed the effect size that we expect to obtain in this group-based randomized trial on detecting a change in the continuous measure of suicide risk (CAT-SS). We assumed there would 200 groups with a total of 1550 Airmen, and over a 6-month period the attrition rate would be 25%. We set a Type I error rate of 5%, power at 80% and assumed a small intra-class correlation ranging from 0.02 to 0.05 on the primary outcome of interest, the suicide scale predictor. Conservatively, we can achieve 80% power in this design with an effect size of 0.15 when the ICC is 0.02 and an effect size of 0.17 when the ICC is 0.05.

All analyses used an intention to treat (ITT) approach to test Wingman-Connect in a realistic setting during training and occurring when a few Airmen-in-Training (AiT) transfer into a different class. Accordingly, each AiTs coding of condition matched that of his or her class (either to Wingman-Connect or stress management) at time of randomization. In all analyses, classes were treated as a clustering variable and random effects were included to account for variability shared by class members, i.e., which meant the trial was analyzed as a cluster randomized trial.[4] Recognizing that classes were originally matched, and that these matches varied in degree (eMethods1), we also conducted sensitivity analyses that used the matched pair as an additional clustering variable and associated random effects were added to the statistical models. All tests were at the 0.05 level and were two-sided.

We tested baseline equivalence of the randomly assigned groups using multi-level analysis of variance (ANOVA) for continuous measures and chi-square tests for two-level categorical variables. To determine whether attrition might have differentially impacted Wingman-Connect and stress management groups, we conducted analyses to compare AiTs who completed follow-up measures with those who were missing at follow-up.

We used multilevel linear and logistic mixed-effects regression models in MPlus version 8.[5] and the R Statistical Package version 4.0.0 to test changes due to training separately on 1-month and 6-month outcomes using models controlling for the baseline version of the chosen dependent variable. Our primary analysis included a single class-level random effect (i.e. a random intercept). We conducted additional analyses accounting for clustering due to pairing of treated and control classes (i.e. random intercept and treatment effects). Because all these analyses yielded comparable statistical conclusions and the models with a single random intercept had lower Bayesian Information Criteria (BIC), we report findings from that analysis. All models also included covariates of sex, age, race, ethnicity, and active duty versus non-active duty, all of which had previously been linked to suicide risk

Results on all mental health problem outcomes were coded so that a negative value indicated more improvement in Wingman Connect compared to stress management, while those involving fitness outcomes were coded so that a positive coefficient represents improved outcomes for Wingman Connect compared to stress management. Tests of significance for main effects of training condition were based on Wald-type tests assessing changes in means adjusted for baseline and other covariates mentioned above. Specifically, for psychological distress measures of CAT-SS and CAT-DEP we used as outcome measures the empirical Bayes estimate of the underlying "severity" latent variable derived from a bifactor model.[3] We tested for Wingman-Connect (versus stress management) impact on health and fitness outcomes in separate models examining baseline to 1-month follow-up (i.e., during technical training) and baseline to final 6-month follow-up (i.e., 2-4 months into first base assignment). Wingman-Connect impact on class and individual AiT risk and protective factors was examined during the technical training period, i.e. one-month follow-up, as by the 6-month they were stationed in different settings. For published scales (e.g., Class Cohesion), outcomes were based on the mean of items within a construct; for CAT dimensional measures (e.g., CAT-SS) the underlying Bayes estimate of severity was used; for a measure of class cohesion and health that combined related scales into a single construct (e.g. psychological distress and fitness), we used single factor analysis that equilibrated loadings across training condition and time; for dichotomized scales (e.g., elevated CAT-DI), two-level binary models are used. To quantify impact for continuous measures, we calculated Cohen's *d* effect sizes ESs[6] adjusted for covariates and including class as a random intercept. For dichotomous outcomes we computed adjusted odds ratios (OR). All tests used a two-sided 0.05 Type I error. To determine if Wingman-Connect varied according to levels of an outcome at baseline, we tested Baseline X Training status interactions by comparing slopes using Wald-type tests that assessed the coefficient's size to its standard error.

Testing the primary theoretical model underlying Wingman-Connect training involved examining mediation effects. Support for this model would be found if AiT perceptions of being connected to a supportive, healthy class mediated the relationship between Wingman-Connect training and suicide risk outcomes. We

© 2020 Wyman PA et al. *JAMA Network Open.*

combined the measures of class relationships and health at each of the two time points (baseline and one month) as single latent factors (i.e., Cohesive Healthy Class Network), fixing the factor loadings to be equivalent across intervention condition. There were slight differences in the loadings across time in the class measure, so we allowed loadings to depend on time; this still provided a legitimate test of impact. Loadings were large for each measure on this latent variable. The fit of the latent variable at both time points was high (RMSEA = .03; CFI = .95); factor loadings for the within factor were above 0.9 with one exception (.56 for bonds to classmates), and lower, as expected, at the class level (.70, .72 and .56). Because training occurred at the second level of class and first level of the Airman's response on the hypothesized mediator and the outcome, these mediational models that involved training condition at baseline, class health at one month, and suicide risk score outcome at 1 month were considered 2-1-1 multilevel mediation models. While we also examined 2-2-1 mediation models, where the one-month mediator was essentially the average score in the class rather than the former individual-level mediator, none of these reached significance, so we have chosen to report only the 2-1-1 mediation models.

© 2020 Wyman PA et al. *JAMA Network Open.*

eTable 2. Baseline Outcomes and Risk/Protective Factors for Full Sample, Wingman-Connect Group and Stress Management Group

Measure, mean (SD)	Full Sample N=1485	Wingman-Connect N=748	Stress Management N=737
Suicide risk[1]	14.01 (13.2)	13.72 (13.0)	14.32 (13.3)
Depression[1]	20.86 (16.3)	20.11 (15.8)	21.60 (16.8)
Occupational impairment[1]	0.29 (0.69)	0.27 (0.65)	0.32 (0.72)
Class cohesion	3.10 (0.59)	3.12 (0.59)	3.09 (0.58)
Class morale	3.73 (0.91)	3.78 (0.91)	3.68 (0.90)
Healthy class norms	2.93 (0.56)	2.95 (0.55)	2.90 (0.57)
Bonds to classmates	2.41 (1.6)	2.53 (1.6)	2.28 (1.5)[2]
Healthy career behaviors	1.71 (0.63)	1.72 (0.61)	1.70 (0.64)
Help seeking acceptability	3.11 (0.56)	3.10 (0.56)	3.12 (0.57)
Maladaptive coping attitudes[1]	1.61 (0.46)	1.61 (0.46)	1.62 (0.47)
Military Functional Impairment[1]	0.29 (0.69)	0.27 (0.65)	0.32 (0.72)
Social	0.60 (0.65)	0.62 (0.64)	0.57 (0.66)
Personal	0.35 (0.57)	0.35 (0.57)	0.35 (0.58)
Loneliness[1]	1.77 (0.75)	1.80 (0.76)	1.73 (0.74)
Anxiety[1]	11.67 (14.9)	10.95 (13.9)	12.40 (15.9)
Anger[1]	0.54 (0.66)	0.53 (0.66)	0.55 (0.65)
Emotional regulation difficulties[1]	1.91 (0.62)	1.91 (0.62)	1.91 (0.61)

[1]Higher scores = greater risk or more problems

[2]Indicates baseline difference between groups randomly assigned to Wingman-Connect or Stress Management conditions ($p<.05$)

© 2020 Wyman PA et al. *JAMA Network Open.*

eFigure 1. Wingman-Connect Impact on Occupational Impairment Indicators in Technical Training

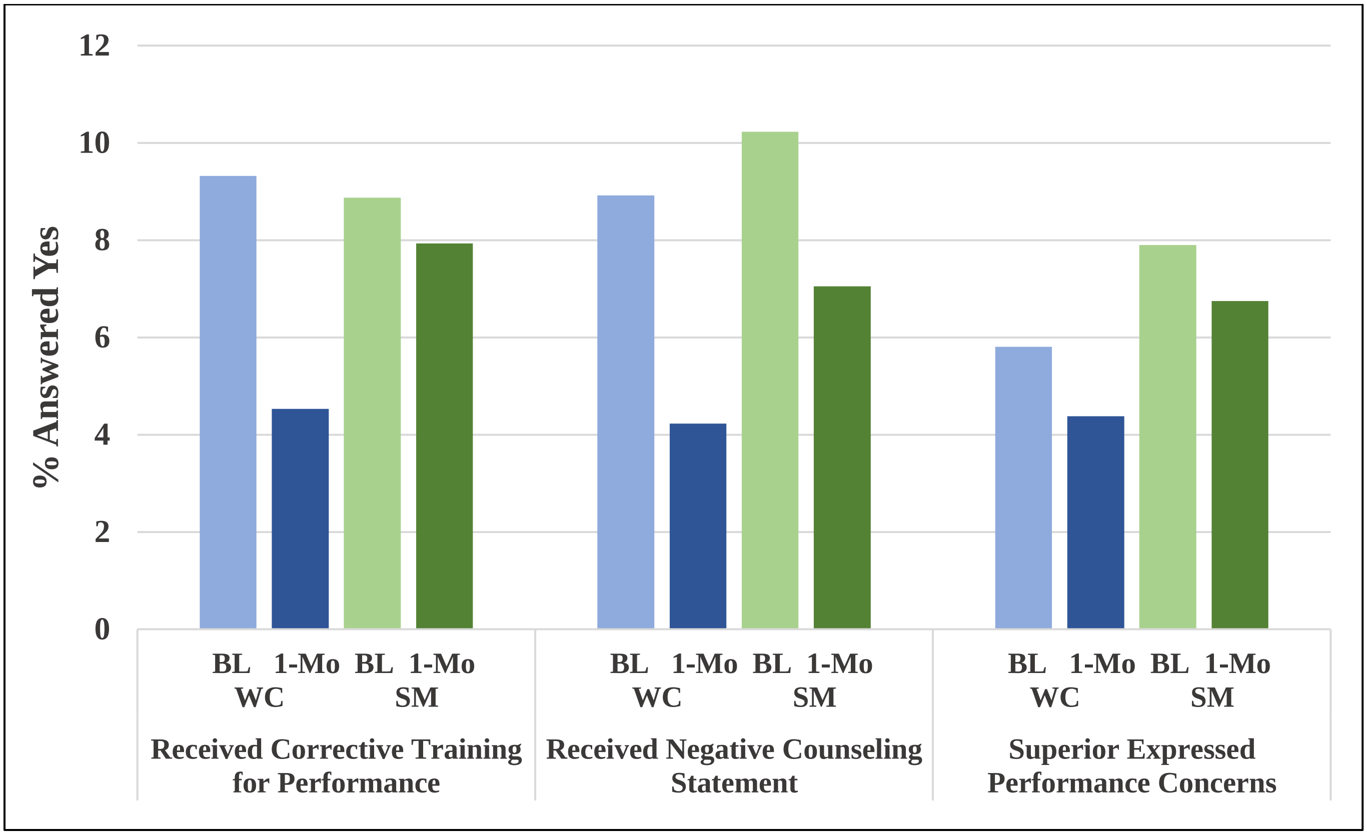

OR=0.51 (95% CI: 0.31,0.82)*   OR=0.50 (95% CI: 0.29,0.86)*   OR=0.66 (95% CI: 0.40,1.12)

**p* < .05. Wingman-Connect trained Airmen had 49% and 50% decreased odds of reporting Corrective Training or Negative Counseling Statements (respectively) in the past 30 days versus Airmen receiving Stress Management Training.

© 2020 Wyman PA et al. *JAMA Network Open.*

eFigure 2. Wingman-Connect Impact on Suicide Severity and Depression Mediated Through Stronger Class Units.

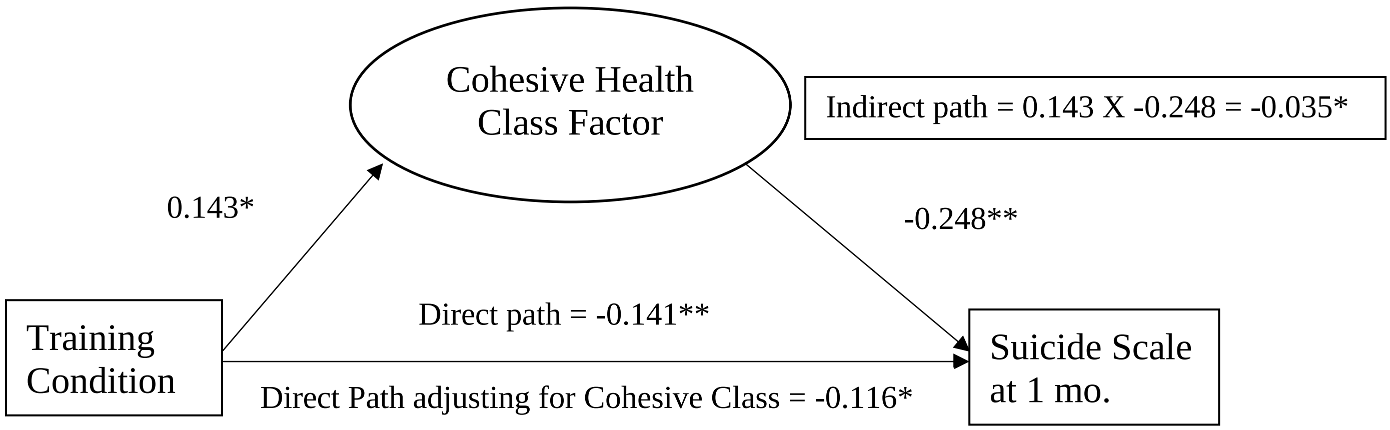

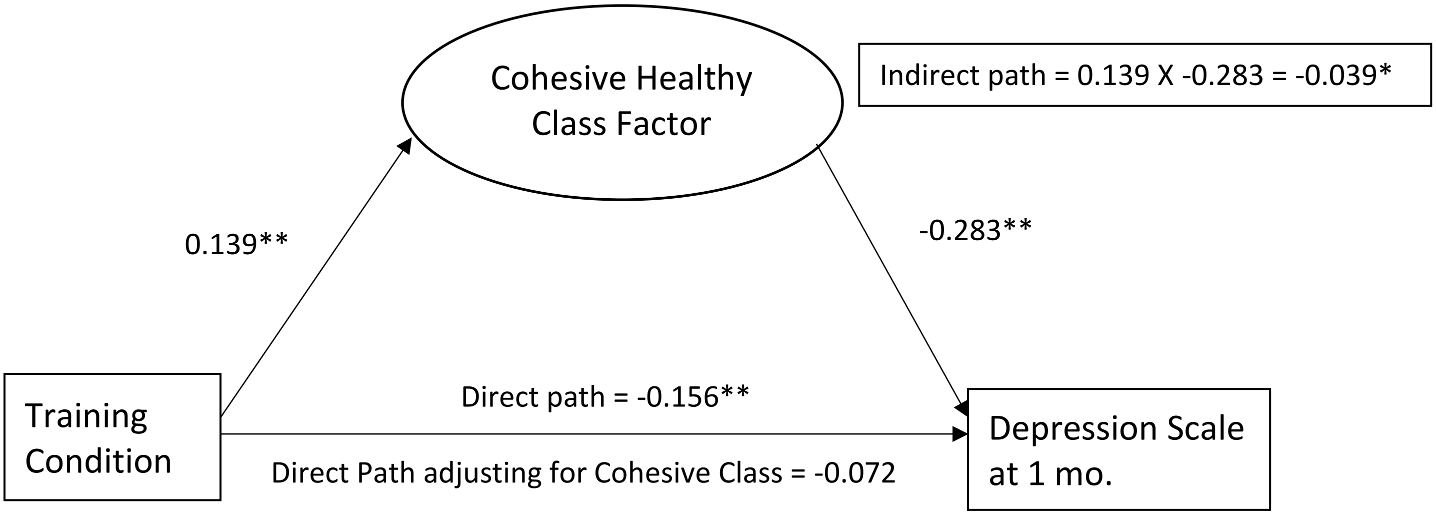

Note: For suicide and depression mediation testing, respectively, suicide (top) or depression (bottom) scores at baseline and covariates are controlled in these models. Both coefficients between training and cohesive healthy class and suicide risk/depression at 1-mo indicate beneficial impact of Wingman-Connect over Stress Management condition.
** p < .01   *p < .05

© 2020 Wyman PA et al. *JAMA Network Open.*

eFigure 3. P-P Plot of Tests of Baseline Equivalence on Behavior Measures

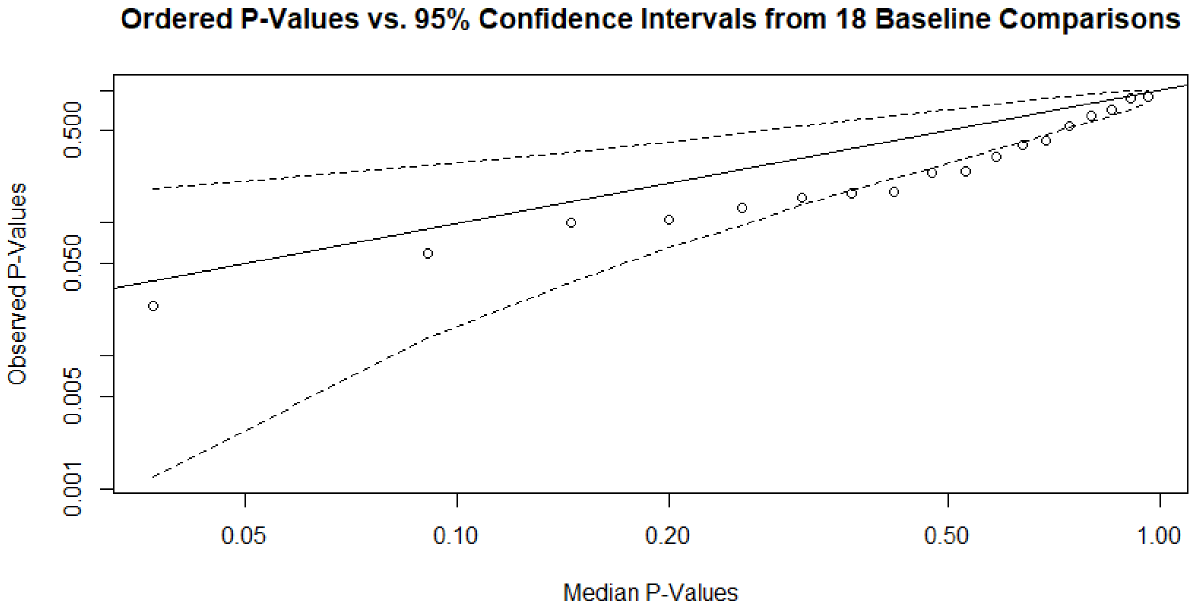

© 2020 Wyman PA et al. *JAMA Network Open.*

eReferences

1. Cross W, Pisani A, Schmeelk-Cone K, et al. Measuring trainer fidelity in the transfer of suicide prevention training. *Crisis.* 2014;35(3):202-212.
2. Vannoy SD, Andrews BK, Atkins DC, Dondanville KA, Young-McCaughan S, Peterson AL. Under reporting of suicide ideation in US Army population screening: An ongoing challenge. *Suicide and Life-Threatening Behavior.* 2017;47(6):723-728.
3. Gibbons RD, Kupfer D, Frank E, Moore T, Beiser DG, Boudreaux E. Development of a computerized adaptive suicide scale: CAT-SS. *Journal of Clinical Psychiatry.* 2017;78(9):1376-1382.
4. Murray D. *Design and analysis of group-randomized trials.* New York, NY: Oxford University Press; 1998.
5. Muthén LK, Muthén BO. *Mplus User's Guide (8th ed.).* Los Angeles, CA: Authors; 2019.
6. Rosenthal R, Cooper H, Hedges L. Parametric measures of effect size. *The handbook of research synthesis.* 1994;621(2):231-244.

© 2020 Wyman PA et al. *JAMA Network Open.*
