# Supplementary material for: Effect of the Wingman-Connect Upstream Suicide Prevention Program for Air Force Personnel in Training: A Cluster Randomized Clinical Trial
Source: JAMA Netw Open. 2020 Oct 21;3(10):e2022532. doi: 10.1001/jamanetworkopen.2020.22532 (PMC7578767; doi:10.1001/jamanetworkopen.2020.22532)
Supplement: Supplement 3. — Data Sharing Statement [file jamanetwopen-e2022532-s003.pdf]

Data Sharing Statement

Wyman. Effect of the Wingman-Connect Upstream Suicide Prevention Program for Air Force Personnel in Training. *JAMA Netw Open*. Published October 21, 2020. 10.1001/jamanetworkopen.2020.22532

Data
Data available: No

Additional Information
Explanation for why data not available: Data from this study are from active duty Air Force personnel. Future decisions to make data available would required authorization from the US Air Force
